# Estimating time-to-total knee replacement on radiographs and MRI: a multimodal approach using self-supervised deep learning

**DOI:** 10.1093/radadv/umae030

**Published:** 2024-11-15

**Authors:** Ozkan Cigdem, Shengjia Chen, Chaojie Zhang, Kyunghyun Cho, Richard Kijowski, Cem M Deniz

**Affiliations:** Department of Radiology, New York University Grossman School of Medicine, New York, NY 10016, United States; Department of Radiology, New York University Grossman School of Medicine, New York, NY 10016, United States; Department of Radiology, New York University Grossman School of Medicine, New York, NY 10016, United States; Center of Data Science, New York University, New York, NY 10011, United States; Courant Institute of Mathematical Sciences, New York University, New York, NY 10012-1185, United States; Department of Radiology, New York University Grossman School of Medicine, New York, NY 10016, United States; Department of Radiology, New York University Grossman School of Medicine, New York, NY 10016, United States

**Keywords:** multimodal learning, time-to-TKR prediction, self-supervised learning, deep learning, knee osteoarthritis

## Abstract

**Purpose:**

Accurately predicting the expected duration of time until total knee replacement (time-to-TKR) is crucial for patient management and health care planning. Predicting when surgery may be needed, especially within shorter windows like 3 years, allows clinicians to plan timely interventions and health care systems to allocate resources more effectively. Existing models lack the precision for such time-based predictions. A survival analysis model for predicting time-to-TKR was developed using features from medical images and clinical measurements.

**Methods:**

From the Osteoarthritis Initiative dataset, all knees with clinical variables, MRI scans, radiographs, and quantitative and semiquantitative assessments from images were identified. This resulted in 895 knees that underwent TKR within the 9-year follow-up period, as specified by the Osteoarthritis Initiative study design, and 786 control knees that did not undergo TKR (right-censored, indicating their status beyond the 9-year follow-up is unknown). These knees were used for model training and testing. Additionally, 518 and 164 subjects from the Multi-Center Osteoarthritis Study and internal hospital data were used for external testing, respectively. Deep learning models were utilized to extract features from radiographs and MR scans. Extracted features, clinical variables, and image assessments were used in survival analysis with Lasso Cox feature selection and a random survival forest model to predict time-to-TKR.

**Results:**

The proposed model exhibited strong discrimination power by integrating self-supervised deep learning features with clinical variables (eg, age, body mass index, pain score) and image assessment measurements (eg, Kellgren-Lawrence grade, joint space narrowing, bone marrow lesion size, cartilage morphology) from multiple modalities. The model achieved an area under the curve of 94.5 (95% CI, 94.0-95.1) for predicting the time-to-TKR.

**Conclusions:**

The proposed model demonstrated the potential of self-supervised learning and multimodal data fusion in accurately predicting time-to-TKR that may assist physicians to develop personalize treatment strategies.

**Abbreviations**

AUC = area under the receiver operating characteristic curve; DESS = sagittal fat-suppressed 3-dimensional dual-echo in steady state; DL = deep learning; KL = Kellgren-Lawrence; MOST = multicenter osteoarthritis study; OA = osteoarthritis; OAI = Osteoarthritis Initiative; RSF = random survival forest; TKR = total knee replacement; TSE = sagittal fat-suppressed intermediate-weighted turbo spin-echo.

**Summary**

Our study developed a predictive model for time-to-total knee replacement using self-supervised deep learning, multimodal data fusion, and survival analysis, demonstrating potential for improving personalized treatment strategies for knee osteoarthritis.

**Key Results**

- The multimodal approach, integrating self-supervised deep learning features with clinical variables and image assessment measurements from multiple modalities, improved the prediction of time-to-total knee replacement (TKR), at any point within a 0- to 9-year timeframe.
- Utilizing multimodal data fusion techniques, our study highlights the effectiveness of advanced artificial intelligence models in improving the prediction of time to TKR, a key outcome in knee osteoarthritis management.
- The proposed model achieved an area under the curve of 94.5 (95% CI, 94.0-95.1).

## Introduction

Osteoarthritis (OA) is a prevalent joint disease, posing a significant global health challenge and resulting in physical disability.^1^ Amongst the various types, knee OA emerges as the most common form of arthritis, profoundly affecting the quality of life for millions worldwide by inducing pain, mobility constraints, and disability.^2^^,^^3^ Despite lacking a definitive cure for knee OA, total knee replacement (TKR) surgery emerges as a plausible intervention during advanced disease stages.^4^ Accurately predicting time-to-TKR may help clinicians make individualized treatment decisions, plan timely interventions, and identify patients at risk, optimizing management strategies for more proactive treatment planning. Predicting time-to-TKR involves using both symptoms reported by patients and findings from imaging modalities.^5–7^ Among these modalities, features on radiographs and MRI,^8–12^ coupled with quantitative and semiquantitative assessments of imaging, have demonstrated associations with knee OA progression, including an elevated risk for TKR.^7^^,^^13–15^ Accurately determining the time-to-TKR is a multifaceted challenge influenced by various factors. This determination hinges not only on disease progression but also on patient-specific considerations including personal preferences, financial constraints, coexisting medical conditions, and overall health status. These factors introduce a level of variability that complicates the prediction of patient timelines.^16–20^ To address the complexities of various factors impacting time-to-TKR, advanced tools are necessary for accurate prediction.

Survival prediction models estimate patient survival likelihood, crucially incorporating also the right-censored data representing event-free duration.^21^ Our study introduced a multimodal approach using self-supervised deep learning (DL) features extracted from radiographs and MRI, allowing us to use unlabeled data and incorporate right-censored data to predict the time-to-TKR. The proposed model predicts the time-to-TKR for each subject, with outputs ranging from 0 to 9 years, representing the estimated time until the TKR surgery. The 9-year timeframe was selected based on the study design of the OAI database, which allows for the observation of each subject's TKR status within the 9-year follow-up period, ensuring that predictions are aligned with the available longitudinal data. We hypothesize that integrating clinical variables, quantitative and semiquantitative assessments from radiographs and MRIs, and DL features into survival models will result in more accurate estimations of time-to-TKR compared to models using a single modality.

## Materials and methods

### Study cohort

The study utilized knee data from two publicly accessible databases – the Osteoarthritis Initiative (OAI)^22^ and the multicenter osteoarthritis study (MOST)^23^ – as well as a retrospective internal testing dataset. The OAI database contains clinical variables, radiographs, MRI examinations, and radiograph and MRI quantitative and semiquantitative image assessment measurements for 4796 subjects aged 45 to 79 years with or at risk for knee OA, evaluated at baseline and follow-ups at 12, 18, 24, 30, 36, 48, 60, 72, and 96 months. The MOST database contains similar clinical information and imaging data for 3026 subjects aged 50 to 79 years with or at risk for knee OA, assessed at baseline and follow-ups at 15, 30, 60, 72, and 84 months. The internal testing dataset was retrospectively built from our hospital system and resulted in radiographs for 164 subjects aged 42 to 91 years with or at risk for knee OA. The OAI and MOST received an ethical approval from the internal review boards at the University of California at San Francisco, Boston University Medical Center, and each individual clinical recruitment site. All participants provided written informed consent. The internal testing dataset was generated using an ethical approval from our hospital system. The study used the OAI dataset for model training and in-distribution testing, while the baseline MOST and internal dataset were used for external testing of the developed models.

**Figure 1.**
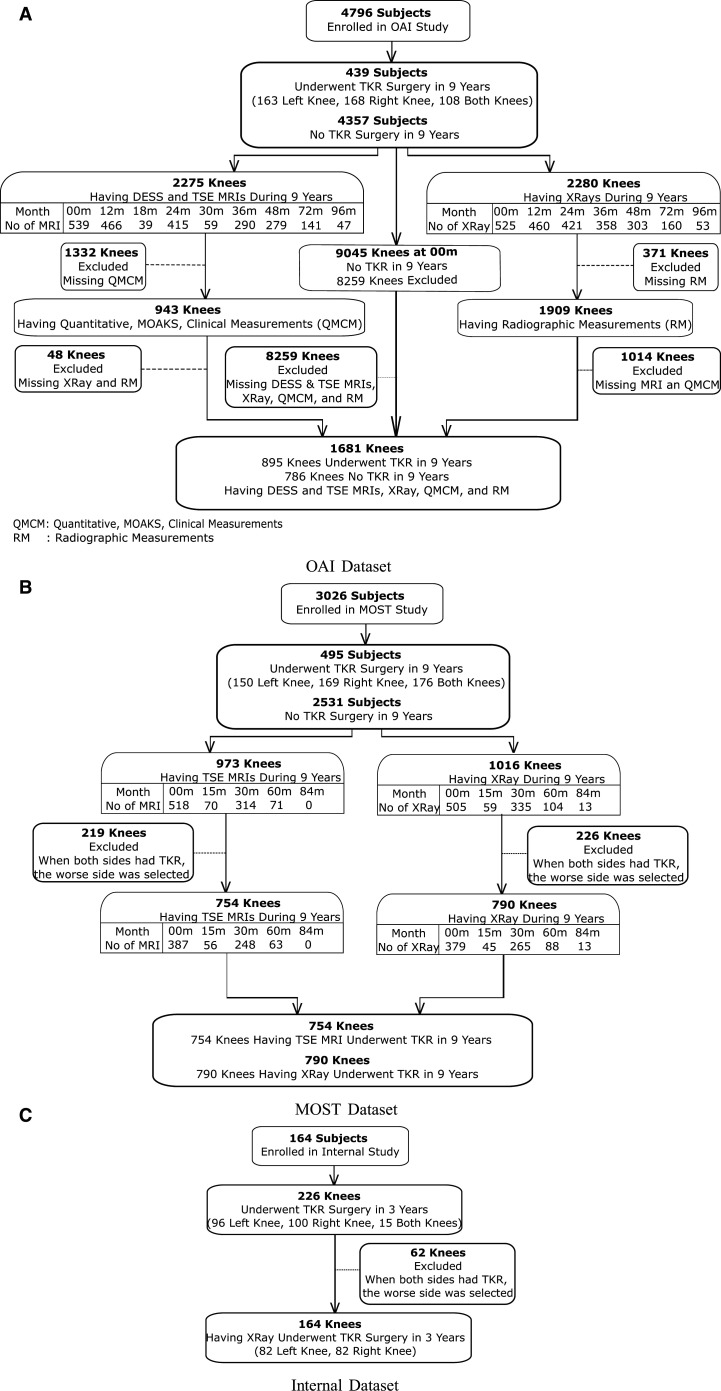
After reviewing clinical, radiographic, and MRI data, as well as clinical, quantitative, and semiquantitative image assessment measurements, the study cohort for predicting time to total knee replacement (TKR) has been determined for (A) Osteoarthritis initiative (OAI), (B) Multicenter Osteoarthritis study (MOST), and (C) internal datasets.

The study cohort in the OAI was evaluated with longitudinal radiographs and MRI examinations from a 3.0-T MRI scanner consisting of sagittal fat-suppressed intermediate-weighted turbo spin-echo (TSE) and sagittal fat-suppressed 3-dimensional dual-echo in steady state (DESS) images. The study cohort in MOST were evaluated with baseline radiographs and TSE image^12^ from a 1.0-T dedicated extremity scanner. In the MOST dataset, when both knees had TKR surgeries during the 9-year follow-up, only the knee in worse condition was selected. In the OAI, each knee that underwent TKR was treated as an independent data point. Follow-up time point data for each patient were treated as independent, separate entries rather than part of a longitudinal study, with each follow-up time considered as year 0 for estimating time-to-TKR. The study cohort selection is summarized in Figure 1, and the specifics of the data are provided in Table 1. Details of the TKR study cohort and data splits are in the Tables S1 and S2.

**Table 1.** Demographic and key clinical and imaging assessment variables of subjects in the Osteoarthritis Initiative (OAI), Multi-center Osteoarthritis study (MOST), and internal study cohorts.

Dataset	OAI		MOST		Internal
Cohort	TKR	Control	TKR	TKR	TKR
No. of knees	895	786	754	790	164
Imaging type	DESS, TSE, X-ray		TSE	X-ray	X-ray
train/validation/test	1239/172/270		-/-/754	-/-/790	-/-/164
Sex					
Male	521	448	205	213	54
Female	374	338	549	577	110
AGE Mean ± SD (range)					
Male	64.7 ± 8.5 (45-83)	62.0 ± 8.9 (45-79)	66.0 ± 7.4 (50-83)	66.5 ± 7.6 (50-83)	64.9 ± 10.3 (42-91)
Female	66.2 ± 8.5 (45-82)	62.5 ± 9.3 (45-79)	67.4 ± 7.2 (50-83)	67.4 ± 7.3 (50-84)	65.8 ± 10.3 (45-88)
Body mass index (mean ± SD)					
Male	29.6 ± 5.2	30.4 ± 5.4	31.0 ± 5.1	31.1 ± 5.1	30.9 ± 5.2
Female	30.0 ± 4.2	29.4 ± 3.9	31.4 ± 5.3	31.6 ± 5.5	31.7 ± 7.3
Race					Not available
Other non-White	18	17	9	9	
White	775	628	710	745	
Black	76	135	35	36	
Asian	26	6	0	0	
KL grade					Not available
0	44	54	72	80	
1	130	93	83	87	
2	260	226	100	101	
3	272	240	250	261	
4	189	173	193	197	
Pain (WOMAC Score)					Not available
0	114	294	127	136	
1-5	385	352	298	310	
6-10	303	107	253	264	
11-15	89	31	73	76	
16-20	4	2	3	4	
OARSI grade					Not available
None	767	661	538	558	
Small	42	33	34	37	
Medium	36	40	63	68	
Large	50	52	50	53	
BML subregions					Not available
0	20	83	0	16	
1-3	303	551	22	28	
4-6	521	139	61	60	
7-9	51	13	22	17	
10-12	0	0	0	1	
Cartilage subregions					Not available
0	7	23	6	2	
1-3	50	222	1	12	
4-6	348	347	24	41	
7-9	391	163	48	39	
10-12	0	0	31	31	

There are 14 cartilage and 15 BML subregions, covering the femoral, tibial, and patellar areas, consistent across the OAI and MOST datasets. A subregion is classified as damaged if the grade is greater than 0.

Abbreviations: BML = bone marrow lesions; DESS = sagittal fat-suppressed 3-dimensional dual-echo in steady state; F = female; internal = internal hospital dataset; M = male; mean = mean of age; KL = Kellgren-Lawrence; OARSI = Osteoarthritis Research Society International; SD = standard deviation; TKR = subjects who underwent total knee replacement over a 9-year follow-up period; Train/Val./Test = number of training; validation, and testing groups; TSE = sagittal fat-suppressed intermediate-weighted turbo spin-echo; WOMAC = Western Ontario and McMaster universities arthritis index.

### Artificial intelligence model

All available quantitative and semiquantitative^24^ image assessment measurements were utilized. Missing measurements in the dataset were imputed using the mean of non-missing values for quantitative data and the mode of nonmissing values for categorical variables.

#### Proposed 2-stage model

In the feature extraction stage, we utilized either supervised or self-supervised DL training. Details of model training are provided in the Supplementary Document, in the section of the proposed 2-stage model.

##### Feature extraction with supervised model

Two separate 3D ResNet18^25^ models were trained using the TSE and DESS MR images and a 2D ResNet18^26^ model was trained using the radiographs. Trained DL models were used to extract the image features from the TKR patients.

##### Feature extraction with self-supervised model

Two separate 3D ResNet18^25^ models were trained on the TSE and DESS MR images using 2 self-supervised frameworks, Med3D^27^ and Twin Class Distribution Estimation.^28^ These models were used to extract representative features from unlabeled 3D knee MR images, including TKR patients and right-censored controls. For radiographs, the ImageNet pretrained Resnet18^26^ model was used to extract features.

##### Feature fusion and multimodal feature selection with Lasso Cox followed by random survival forest modeling

**Figure 2.**
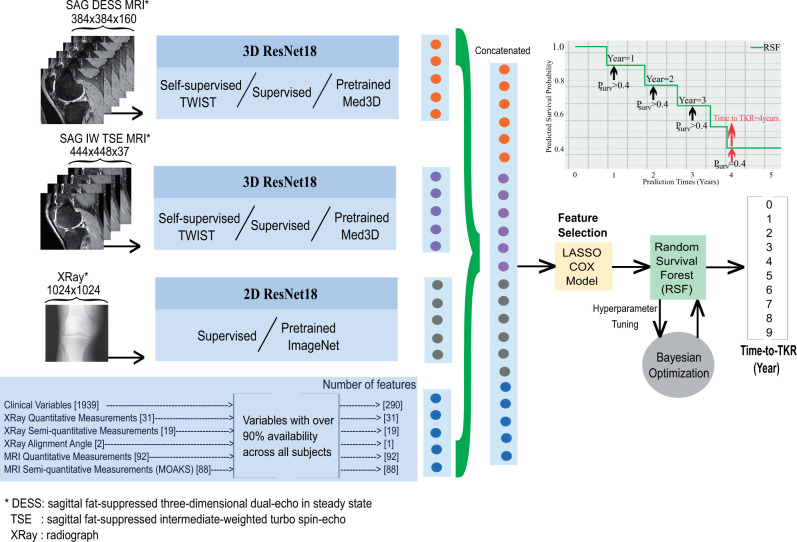
The flowchart of the proposed approach for predicting time to total knee replacement (TKR).

**Figure 3.**
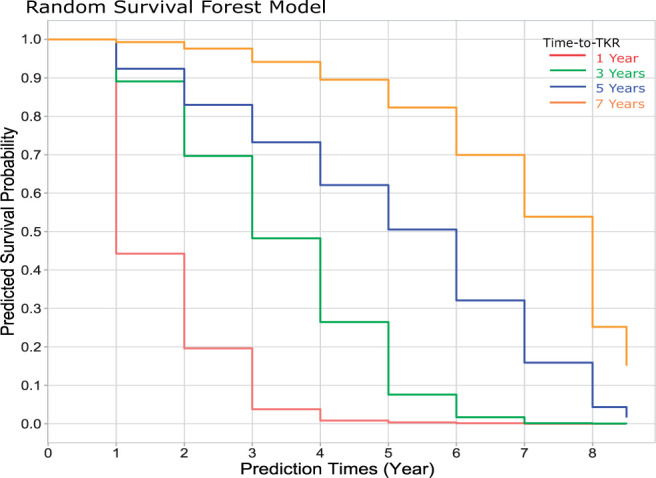
Predicted survival probabilities of patients who underwent total knee replacement (TKR) surgery at 1 year, 3 years, 5 years, and 7 years.

DL features were extracted from the output of the last pooling layer of the supervised/self-supervised models and concatenated with features from multimodal data prior to applying feature selection. For feature selection, a Lasso method was applied to the Cox regression model.^5^ To address violations of proportional hazards often encountered in high-dimensional data, unlike Cox proportional hazard models and their variations, the random survival forest (RSF) model was employed for time-to-TKR prediction.^21^ The time-to-TKR was determined as the latest year in which the survival probability exceeds the threshold of 0.4. This threshold was selected based on the best validation accuracy. The flowchart of the proposed models is presented in Figure 2 and further detailed in Table S3, with comparison of RSF modelling with other common approaches. Figure 3 provides an illustrative example of predicted survival probabilities for patients who underwent TKR surgery at 1, 3, 5, and 7 years.

#### End-to-end trained model for external testing and comparison with the proposed approach

##### Model training and testing with the OAI dataset

For the prediction of time-to-TKR in 9 years, 2D and 3D ResNet18^26^ DL models were trained with image scans, along with clinical variables and image readings from the OAI dataset, using Kullback-Leibler divergence as the loss function.

##### Testing with MOST and internal datasets

The trained DL models were applied to both the MOST and an internal testing dataset. Model performance could be evaluated in the MOST and internal testing datasets using DL features only because not all clinical variables and image readings were available in the MOST and internal datasets.

### Evaluation metrics

The concordance index is calculated as the ratio of concordant pairs to total pairs, with values closer to 1 indicating better predictive performance. It evaluates a model's accuracy in predicting time-to-TKR by assessing “concordant pairs,” where the patient with a shorter predicted time-to-TKR undergoes surgery earlier than a patient with a longer predicted time. “Total pairs” refers to all possible pairs where 1 patient's TKR time is known to be earlier than or equal to another’s.

Accuracy is calculated as the proportion of correctly predicted subjects to the total number of subjects.

Area under the curve (AUC) was calculated for each prediction year from 0 to 9 in the survival analysis. For each year, the model evaluated its ability to distinguish between patients who underwent TKR and those who did not. The macro-AUC was determined by averaging the AUC values from each prediction year.

### Statistical analysis

The Wilcoxon signed-rank test was used to evaluate differences in performance between the different models. Statistical significance was defined as *P* < .05. Details of the software used in statistical analysis are provided in the Supplementary Document.

## Results

### Participant characteristics

The study included data from several datasets. In 895 knees from the OAI dataset that underwent TKR over 9 years, there were 109 males (mean age: 64.2 ± 9.3) and 170 females (mean age: 62.4 ± 8.1). Among 786 right-censored control knees from the OAI dataset, there were 322 males (mean age: 62.1 ± 9.3) and 432 females (mean age: 61.9 ± 9.3). For TSE MRI scans and radiographs from the MOST dataset, there were 106 males (mean age: 65.1 ± 7.4) and 281 females (mean age: 65.6 ± 7.1), and 103 males (mean age: 65.1 ± 7.4) and 276 females (mean age: 65.5 ± 7.1), respectively. Additionally, from the internal hospital dataset, there were 54 males (mean age: 64.9 ± 10.3) and 110 females (mean age: 65.8 ± 8.8).

#### Proposed 2-stage model

Table 2 presents the performance of several 2-stage models in estimating time-to-TKR.

**Table 2.** Comparison of supervised and self-supervised models for estimating time-to-total knee replacement.

Testing datasets	Image feature extraction model (details in sections 1.1a and 1.1b)	Fused multimodal features as input to random survival forest model (details in Section 1.2)	Number of features	Accuracy %	C-index %	Area under the curve (95% CI)	Integrated Brier score	*P* value (Wilcoxon test)
	Supervised (1.1a)	DESS + TSE + X-ray	1024	63.7	67.6	76.5 (73.7-79.3)	0.106	^a^
		DESS + TSE + X-ray + clinical variable + image measurement	1545	70.7	71.8	78.4 (73.8-83.0)	0.098	*<.*001^a^
		Lasso Cox on DESS + TSE + X-ray + clinical variable + image measurements	91	73.2	76.2	85.1 (82.2-88.0)	0.09	*<.*001^a^
	–	Lasso Cox on clinical variable + image measurement	90	63	72.3	84.0 (81.3-86.7)	0.09	*<.*001^b^
OAI	Self-supervised Med3D	DESS + TSE	512	41.5	56.0	71.9 (70.5-73.2)	0.118	.002^b^
	(1.1b)							
	Self-supervised	DESS + TSE	512	42.2	69.8	76.5 (75.6-77.4)	0.106	.002^b^
	Twin class distribution estimation	DESS + TSE + X-ray	1024	52.6	73.8	81.5 (80.6-82.4)	0.094	^b^
	(1.1b)	DESS + TSE + X-ray + clinical variable	1314	58.9	78.9	86.5 (86.1-86.9)	0.081	.012^b^
		DESS + TSE + X-ray + clinical variable + image measurement	1545	74.4	85.0	93.9 (93.1-94.6)	0.058	*<.*000^b^
		Lasso Cox on DESS + TSE + X-ray + clinical variable + image measurement	**95**	**75.2**	**85.3**	**94.5 (94.0-95.1)**	**0.055**	*<.*001^b^

Accuracy (number of correctly predicted subjects/all subjects). Boldface indicates the proposed model.

Abbreviations: AUC = area under the curve (macro-AUC over 9 years); C-Index (%) = concordance index; DESS = sagittal fat-suppressed 3-dimensional dual-echo in steady state; IBS = Integrated Brier score-IBS (mean IBS over 9 years); image measurements = quantitative and semiquantitative image assessment measurements; OAI = osteoarthritis initiative; TSE = sagittal fat-suppressed intermediate-weighted turbo spin-echo.

a, b = Reference model for Wilcoxon statistical test.

##### Features extracted with supervised model

The model using concatenated image data alone was used as the reference model for statistical analysis. Adding clinical variables and image measurements to concatenated image data improved the prediction performance. Applying Lasso Cox to the combined multimodal features further enhanced performance, resulting in statistically significant improvements compared to the reference model. Both models yielded statistically significant results when compared to the reference model.

##### Features extracted with self-supervised model

The model using concatenated features from DESS and TSE MRI sequences and radiographs, outperformed the model with only DESS and TSE MRI features and the pretrained Med3D. Incorporating clinical variables and image assessments further enhanced model performance. Applying Lasso Cox selection to the combined multimodal features yielded the best performance for predicting time-to-TKR. All models provided statistically significant results compared to the reference model, which used the concatenation of radiographs and TSE and DESS MRI images alone.

The proposed model, which incorporates feature selection with multimodal data, utilized 95 selected features for prediction. These features included various clinical factors such as age, body mass index, pain level, quality of life, difficulty standing from a seated position or getting in and out of a car, as well as the use of hyaluronic acid or steroid injections, medications for pain management, and health care coverage. Radiographic readings included the Kellgren-Lawrence grade and joint space narrowing (Osteoarthritis Research Society International grades) in both the lateral and medial compartments. Additionally, MRI readings encompassed cartilage thickness in the medial tibia, cartilage morphology across all measured areas, bone marrow lesion size in the lateral tibia, the number of bone marrow lesions in the medial anterior femur, Osteoarthritis Research Society International tears, and lateral meniscal extrusion. The details of the selected features, confusion matrices, and ablation studies are provided in Tables S3-S5 and Figures S1-S3.

### End-to-end trained model

Table 3 presents the performance of supervised models across OAI, MOST, and internal testing datasets.

**Table 3.** Comparison of supervised models for estimating time-to-total knee replacement.

Testing datasets	Models (details in Sections 1 and 2)	Fused multimodal features as input to models	Number of features	Accuracy %	C-Index %	AUC (95% CI)		Integrated Brier Score	*P*-value (Wilcoxon test)
OAI	End-to-dnd	TSE	–	58.5	58.1		–	–	–
MOST	Training	TSE	–	47.8	51.0		–	–	–
OAI	(2.1a & 2.1 b)	X-ray	–	62.8	60.4		–	–	–
MOST		X-ray	–	48.5	50.2		–	–	–
Internal		X-ray	–	55.5	53.5		–	–	–
OAI		DESS + clinical variables + image measurement	–	64.3	65.9		–	–	^a^
OAI		TSE + clinical variable	–	59.2	60.2		–	–	^b^
OAI		X-ray + clinical variable + image measurement	–	52.4	57.6		–	–	^c^
	Two-Stage Method (1.1a & 1.2)	DESS + clinical variable + image measurement	685	66.2	69.1	75.3	(69.7-80.9)	0.107	.062^a^
		TSE + clinical variable	505	56.7	59.2	58.7	(54.4-63.1)	0.119	.073^b^
OAI		X-ray + clinical variable + image measurement	803	61.8	67.5	67.3	(57.0-77.5)	0.110	.018^c^
		Lasso Cox on DESS + clinical variable + image measurement	66	66.9	72.3	78.1	(74.3-81.9)	0.102	.045^a^
		Lasso Cox on TSE + clinical variable	25	63.7	65.2	68.3	(63.3-73.4)	0.106	.046^b^
		Lasso Cox on XRay + Clinical Variable + Image Measurement	77	63.7	68.1	76.5	(72.7-80.4)	0.105	.012^c^

Accuracy (number of correctly predicted subjects/all subjects).

Abbreviations: AUC = area under the curve-AUC (macro-AUC over 9 years); C-Index (%) = concordance index; DESS = sagittal fat-suppressed 3-dimensional dual-echo in steady state; IBS = Integrated Brier score (mean IBS over 9 years); image measurements = quantitative and semiquantitative image assessment measurements; MOST = Multicenter Osteoarthritis Study; OAI = Osteoarthritis Initiative; TSE = sagittal fat-suppressed intermediate-weighted turbo spin-echo.

a, b, c = reference model for Wilcoxon statistical test.

***OAI testing dataset***. Two-stage models with Lasso Cox feature selection consistently outperformed end-to-end DL models for predicting time-to-TKR across all modalities, incorporating related nonimaging features. Notably, the 2-stage models without Lasso Cox feature selection showed statistically significant improvement only for radiographs (Accuracy (ACC): 61.8%, P = .018) but not for TSE (ACC: 56.7%, P = .073) and DESS (ACC: 66.2%, P = .062) MR images.

***MOST and internal testing datasets***. The DL model using radiographs provided an estimation accuracy of 62.8% for the OAI testing dataset, which dropped to an estimation accuracy of 48.5% in the external MOST testing dataset and 55.5% in the internal dataset, respectively. Likewise, the DL model using TSE image provided an estimation accuracy of 58.5% for the OAI testing dataset, which dropped to an estimation accuracy of 47.8% in the MOST testing dataset.

The performance of our 2-stage model is compared to the existing methods, as shown in Table 4.

**Table 4.** Comparing the performance of existing methods applying them to the datasets used in this study: the differences in the number of features arise from 2 factors: (1) missing values for more than 50% of knees and (2) enrollment variables (race, sex, history of knee arthroscopy) being identical across all visits.

Research work	Random survival Forest model input (number of features^a^)	Number of features	Accuracy %	C-Index %	AUC (95% CI)	*P* value of Wilcoxon test
Jamshidi et al.^5^	MOAKS + radiographic + clinical variable (3)	3	56.7	71.5	81.4 (79.9-82.9)	*<*.001
Heisinger et al.^6^	Radiographic + clinical variable (14)	14	58.1	73.1	85.3 (82.5-88.1)	*<*.001
Mahmoud et al.^7^	Radiographic + clinical fariable (45)	31	45.2	62.7	69.9 (68.0-71.7)	*<*.001
Liu et al.^30^	Radiographic + clinical fariable (9)	6	60	73.5	85.2 (83.8-86.6)	*<*.001
**Our Study**	**Proposed model**	**95**	**75.2**	**85.3**	**94.5** (94.0-95.1)	^a^

Accuracy (number of correctly predicted subjects/all subjects).

Abbreviations: Area under the curve-AUC = macro-AUC over 9 years; C-Index (%) = concordance index; Integrated brier score-IBS = mean IBS over 9 years; MOAKS = MRI osteoarthritis knee score; Number of Features* = number of features used in the original study; radiographic = quantitative, semiquantitative, and alignment measurements of radiograph images.

a = Reference model for Wilcoxon statistical test.

**Figure 4.**
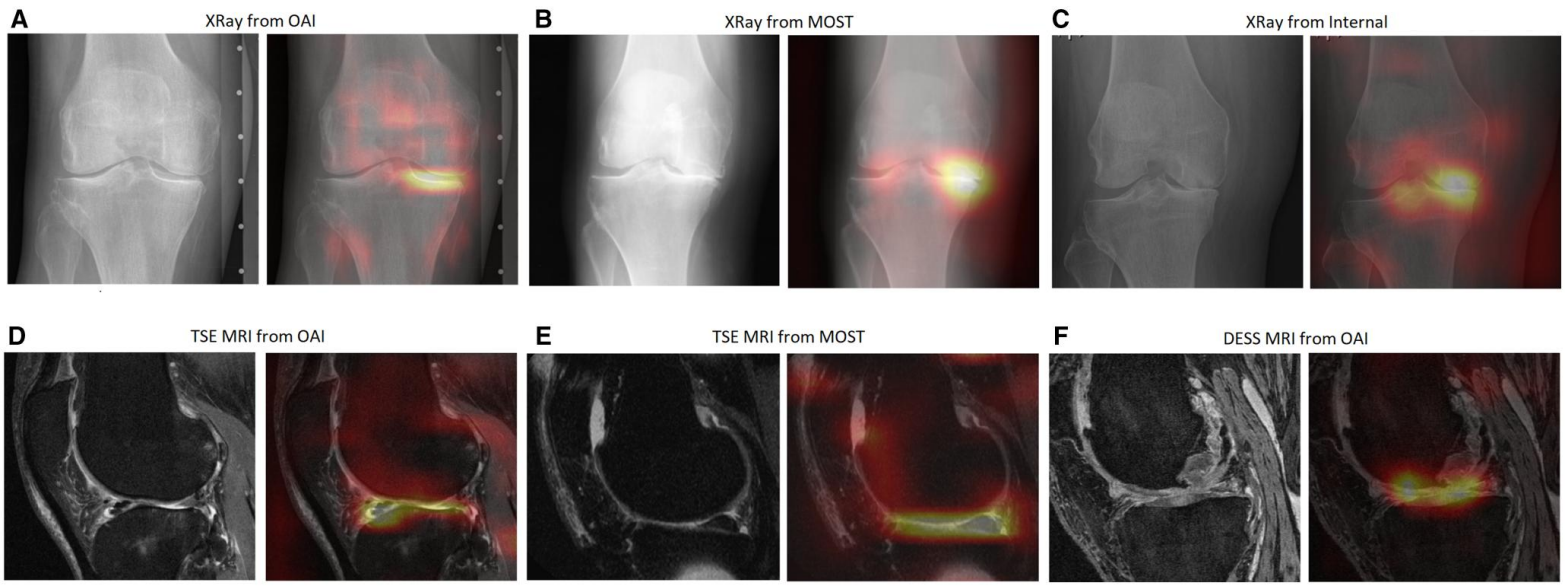
Representative examples and saliency maps of deep learning models using (A) radiographs from the Osteoarthritis initiative (OAI) dataset, (B) radiographs from the Multicenter Osteoarthritis study (MOST) dataset, (C) radiographs from the internal dataset, (D) TSE MRI from the OAI dataset, (E) TSE MRI from the MOST dataset, and (F) DESS MRI from OAI dataset. The subjects from the OAI, MOST, and internal datasets underwent total knee replacement in 3, 6, and 1 year, respectively. DESS = dual-echo in steady state, TSE = turbo spin-echo.

The saliency maps of the DL models using radiographs and MRI are shown in Figure 4 for the OAI, MOST, and internal datasets.

## Discussion

Our study demonstrated that integrating clinical variables, along with quantitative and semi-quantitative assessments from radiographs and MRI scans, as well as the DL features extracted from these imaging modalities, into predictive models resulted in higher accuracy in estimating the time-to-TKR compared to models analyzing DL features individually. The performance of using self-supervised pretrained DL model and right-censored data provided better prediction performance compared to supervised DL model using only the subjects underwent TKR in a 9-year time frame. The proposed multimodal model using self-supervised learning correctly ranked 85.3% of subjects based on their TKR risk, with subjects at a higher risk more likely to require surgery sooner.

Recent studies have explored machine learning models to predict TKR using clinical variables and image assessments.^5–7^^,^^29^ Jamshidi et al^5^ focused on TKR binary classification with baseline clinical data and imaging assessments. Although Heisinger et al^6^ predicted TKR using clinical variables and the Kellgren-Lawrence (KL) grade over a 4-year period, Liu et al^29^ analyzed TKR prediction using clinical variables alone and combined with KL grade over a 5-year follow-up and Mahmoud et al^7^ used clinical variables and KL grade to predict TKR over 2 and 5 years. However, our study uses self-supervised DL features from radiographs and MRI scans in AI models to predict time-to-TKR over a 9-year period. Compared to existing literature, our model demonstrates better performance in predicting time-to-TKR, attributed to the integration of diverse features from multimodal data. The fusion of imaging, clinical, and image features offers a promising approach to address the limitations of each modality when used alone, allowing for a more comprehensive analysis of complementary information.

Our proposed model incorporated all available features from the OAI database to identify the most discriminative predictors of time-to-TKR, including previously studied clinical factors, radiographic readings, and MRI-based measurements.^5–7^^,^^9^^,^^29–33^ The features selected by our model align with prior research, underscoring the importance of considering a comprehensive set of clinical, radiographic, and MRI-based factors in predicting time-to-TKR. Of the 95 selected features, 30 were derived from radiographic and MRI scans, highlighting the valuable insights that these modalities provide. Clinical factors included age,^7^^,^^9^^,^^29^ body mass index,^6^^,^^7^^,^^9^^,^^29^ pain level,^5–7^^,^^9^^,^^29^ quality of life,^6^ and difficulty standing from a seated position or getting in and out of a car,^7^ along with the use of hyaluronic acid or steroid injections^5^^,^^7^ and medications for pain management.^6^ Radiographic readings comprised the KL grade^5–7^^,^^9^^,^^29^ and joint space narrowing (Osteoarthritis Research Society International grades) in both lateral and medial compartments.^5^^,^^9^ Additionally, MRI readings included cartilage thickness in the medial tibia,^9^^,^^30^^,^^31^ cartilage morphology across all measured areas,^9^^,^^30^^,^^31^ bone marrow lesion size in the lateral tibia,^32^^,^^33^ the number of bone marrow lesions in the medial anterior femur,^32^^,^^33^ anterior cruciate ligament tears,^32^ and lateral meniscal extrusion.^32^^,^^33^ The integration of these features with image data resulted in improved prediction performance compared to using clinical variables and image measurements alone.

The performance of 2-stage model was better than that of end-to-end trained DL model for time-to-TKR prediction. Training a DL model end-to-end with both multimodal images and clinical data are resource-intensive and could suffer from GPU memory constraints. The 2-stage approach leverages the strengths of both ResNet18 and RSF models by separating feature extraction from prediction, which reduces the overall model complexity compared to end-to-end training. RSF is specifically designed for survival analysis and effectively handles censored data. Additionally, the regularization effects of Lasso Cox feature selection also contribute to a more robust model by eliminating irrelevant features and mitigating possible overfitting.

Knee OA is a slowly progressing disease, and currently, standardized clinical assessment methods for directly predicting time-to-TKR are not available. Therefore, external testing for generalization from a broader population was limited. Our DL models analyzing features extracted from radiographs and the TSE image showed a drop in diagnostic performance when evaluated using an external testing dataset in MOST and internal testing datasets. This highlights the challenges for the widespread application of DL models analyzing features extracted from baseline imaging studies in clinical practice, which may be influenced by variability in imaging equipment and protocols across institutions. The drop in diagnostic performance when evaluated using an external testing dataset was also observed in the existing literature.^7^^,^^29^ Future work should focus on AI models less sensitive to image variations and training methods using heterogeneous image datasets to improve DL model performance and generalizability.

Our study has limitations. We used 2 knees of the same patient, which may be correlated, but treating them as separate data points increases the sample size, boosting statistical power and capturing individual knee variability. Although some clinical data, such as general health indicators, apply to both knees, each knee has distinct measurements and images, so we treated them independently in our model since their condition and progression can vary. Due to current GPU memory constraints, end-to-end multimodal training with all imaging data using the 3D convolutional neural networks was hindered. The data from the OAI, MOST, and internal databases, primarily composed of older, overweight, and Caucasian subjects, were used. Thus, model generalizability to more age, body mass index, race, and ethnic diverse subject populations needs to be further investigated. Additionally, the MOST database utilizes a dedicated 1.0-T extremity MRI, which is unlikely to have broad applicability in nonspecialist centers, further limiting the generalizability of the model. Our imputation method, using the mean for quantitative and the mode for categorical data, is less complex and time-efficient but may introduce bias, especially with nonrandom missing data. Advanced methods, like multiple imputation or machine learning approaches such as k-nearest neighbors or random forest, may offer better accuracy by preserving data patterns and variability. We will explore these alternatives in future work to improve robustness. Future work will also focus on integrating radiograph and MRI sequences with tabular data for end-to-end training. Additionally, we will explore the integration of the proposed model into clinical workflows and its adaptability in different medical environments.

## Author contributions

Ozkan Cigdem (Conceptualization, Formal analysis, Investigation, Methodology, Software, Validation, Visualization, Writing—original draft, Writing—review & editing), Shengjia Chen (Conceptualization, Formal analysis, Methodology, Software, Validation, Writing—review & editing), Chaojie Zhang (Conceptualization, Methodology, Software, Writing—review & editing), Kyunghyun Cho (Conceptualization, Investigation, Supervision, Writing—review & editing), Richard Kijowski (Conceptualization, Investigation, Methodology, Supervision, Writing—review & editing), and Cem Deniz (Conceptualization, Formal analysis, Funding acquisition, Investigation, Methodology, Project administration, Resources, Supervision, Validation, Visualization, Writing—review & editing)

## Supplementary material

Supplementary material is available at *Radiology Advances* online.

## Funding

This study is supported in part by the National Institutes of Health (R01 AR074453).

## Conflicts of interest

Please see ICMJE form(s) for author conflicts of interest. These have been provided as supplementary materials.

There is no conflict of interest to declare for any of the authors.

## Data and code availability

We utilized 3 distinct datasets: OAI, MOST, and our internal dataset. The OAI and MOST datasets are accessible at (https://nda.nih.gov/oai) and (https://most.ucsf.edu/multicenter-osteoarthritis-study-most-public-data-sharing), respectively. Our internal dataset was employed for retrospective testing in this study.

The source code for this study is available at https://github.com/denizlab/2024_RadiologyAdvances_time2TKR

## Supplementary Material

umae030_Supplementary_Data
