# Supplementary material for: Estimating time-to-total knee replacement on radiographs and MRI: a multimodal approach using self-supervised deep learning
Source: Radiol Adv. 2024 Nov 15;1(4):umae030. doi: 10.1093/radadv/umae030 (PMC11687945; doi:10.1093/radadv/umae030)
Supplement: umae030_Supplementary_Data [file umae030_Supplementary_Data.pdf]

# Supplementary Document

## Materials and Methods

### Study cohort

All knees from the OAI were utilized in cohort selection. We excluded 2,765 knees underwent TKR in 9-years due to missing MRI image readings (n=1,332 knees), missing radiograph image readings (n=371 knees) or missing both radiograph and MRI images and readings (n=1,014 and n=48 knees). Similarly, we excluded 9045 knees that did not undergo TKR in 9-years due to missing clinical and image assessment measurements. A total of 1,681 knees (895 knees with TKR within 9-years and 786 knees as right-censored controls) had complete clinical variables, radiograph and MRI image assessment measurements [24]. The study cohort is summarized in Figure 1 and the baseline gender and age of study cohorts are provided in Table 1 in the main paper. The dataset was partitioned into 1,239 training, 172 validation, and 270 testing data. The training, validation, and testing data splits were done at the subject level so that all follow-up data associated with the same subject was included in a single split. The details of the study cohort that underwent TKR and data splits are presented in Table S1.

### Artificial intelligence model

Of the 1,939 baseline clinical variables, 290 were available for over 90% of the subjects

in the OAI database. A two-stage training approach was utilized: feature extraction and feature fusion. The extracted features were then concatenated with clinical variables and image readings to train a random survival forest (RSF) model, aimed at predicting time-to-TKR. Through using RSF's bootstrap aggregation and random feature selection, the need for multiple evaluations with different dataset splits was eliminated. This approach was chosen over traditional cross-validation due to the computational cost associated with cross-validation and hyperparameter tuning in DL model training. A two-stage training approach

was utilized: (1: feature extraction) DL features were extracted from radiographs and MRI using either supervised or self- supervised DL models. (2: feature fusion).

**1. Proposed two-stage model**

A two-stage model contains a feature extraction stage followed by a fusion stage and feature selection as described below.

***1a. Feature extraction with supervised model***. Models were trained using the Kullback–Leibler divergence loss, which ensured that the output of the model had a distribution similar to the normal distribution of the labels. The labels representing time-to-TKR were mapped to a normal distribution (discretized into 30 bins) with a variance of 4 and a mean equal to the label. Predictions were calculated based on the area under the predicted distribution. The best model for each image type was selected based on the highest accuracy achieved on the validation data.

***1b. Feature extraction with self-supervised model***. The TWIST framework employed a Siamese network to generate twin class distributions of two augmented MR images. A combination of three loss terms was introduced to encourage the model to extract distinct features of different MR images: (1) the consistency term enforced the class distributions of two augmented views to be consistent, (2) the sharpness term enforced each sample distribution to be sharp, making each sample have a deterministic assignment, and (3) the diversity term enforced different samples to be diversely distributed to different classes. Trained TWIST models were used to extract features from the study cohort involving TKR patients and right-censored controls.

***1.2. Feature fusion and multi-modal feature selection with Lasso Cox followed by RSF modelling***.

RSF, an ensemble nonparametric method, benefits from bootstrap aggregation and random feature selection at each node, leading to lower generalization error. The hyperparameters of the

RSF model were optimized using the Bayesian optimization algorithm and tuning was performed by minimizing the integrated Brier score. The RSF model's output consisted of predicted survival probabilities, indicating the likelihood of not undergoing TKR surgery, for each subject over a 9-year period.

**2. End-to-end trained model for external testing and comparison with the proposed approach**

***2.1a. Model training and testing with OAI dataset.***

In the OAI dataset, quantitative image assessments are available for DESS MR images and radiographs. DL model end-to-end training was conducted separately using DESS MR images or radiographs, along with associated clinical variables and image readings. For the TSE MRI sequence, the DL model was trained end-to-end using TSE MR images and clinical variables. The clinical variables and image readings were concatenated with the image features from Resnet18 model before being fed into the final fully connected layer. The output of the trained supervised models was used as the prediction of time-to-TKR.

To ensure a direct comparison with existing literature, the clinical variables and image measurements identified in prior studies were employed with our dataset. However, some features were excluded due to missing values in over 50% of the knees. The remaining variables were utilized for analysis.

**Model Training**

An ensemble AI model combining Resnet18 and RSF models was trained to predict time-to-TKR surgery within a 9-year timeframe. Both Resnet18 and RSF models were trained and evaluated using the same data split: 70% for training, 10% for validation, and 20% for testing. Horizontal flipping and random crops were used for data augmentation. To improve model generalizability, random cropping of input image size to 768x768,

300x300x37, and 300x300x160 was implemented for the radiograph, TSE, and DESS models, respectively. 3D Resnet18 DL models using TSE and DESS MRI sequences were trained for 200 epochs, while 2D Resnet18 model using radiograph was trained for 500 epochs. Adam optimizer was used with a learning rate and a weight decay of 10-4. The model with the best validation accuracy was selected as and a weight decay of 10-4. The model with the best validation accuracy was selected as the best model. The second last layer of Resnet18 DL model, the output of global max pooling layer before fully connected one provided 512 features for each image modality [1].

**Self-Supervised Learning Framework TWIST**

In this study, the self-supervised learning framework TWIST was used to extract representative features from unlabeled 3D knee MR images. The TWIST model took two augmented views from the knee MR images and generated two pseudo-class distributions. The two pseudo-class distributions were regularized to be close in the TWIST loss, enforcing the extracted features from two different augmented views to be consistent. Meanwhile, the pseudo-class distributions of different knee MR images from the same batch were regularized to be sharp and diverse, enforcing the extracted features from different samples to be unique. The loss function of the TWIST model is shown in equation 1, where the pseudo-class distributions of two augmented views in the same batch (batch-size = $B$) are defined as $P^1$ and $P^2$. $D_{KL}$ denotes the Kullback-Leibler divergence between two probability distributions. $H$ denotes the Entropy of a specific probability distribution. $\alpha$ and $\beta$ are hyper-parameters to balance different loss terms.

[1]E. Hedayati, H. Rajamohand, L. Zhou, K. Cho, G. Chang, R. Kijowski, C. M. Deniz, Estimating time-to-total knee replacement surgery using deep learning, in: Proceedings of the 2023 ISMRM & ISMRT Annual Meeting & Exhibition, Toronto, Canada, 2023.

The loss function includes three terms:

(1) the consistency term enforces the class distributions of two augmented views to be consistent,

(2) the sharpness term enforces each sample distribution to be sharp,

(3) the diversity term enforces different samples to be diversely distributed to different classes.

$$L(P^1, P^2) = \frac{1}{2B}\sum_{i=1}^{B}\left(D_{KL}(P_i^1 \parallel P_i^2) + D_{KL}(P_i^2 \parallel P_i^1)\right) + \frac{\alpha}{2}\sum_{k=1}^{2}\frac{1}{B}\sum_{i=1}^{B}H(P_i^k) - \frac{\beta}{2}\sum_{k=1}^{2}H\left(\frac{1}{B}\sum_{i=1}^{B}P_i^k\right) \tag{1}$$

The Lasso Cox feature selection model used the cv.glmnet function in R with subject-specific cross-validation (knees from the same patient were grouped together) to select the optimal regularization parameter that minimized the mean cross-validated error. The same training and validation data cohorts utilized in the DL Resnet models were used in the RSF model. The hyperparameters of the RSF model were optimized using the Bayesian optimization algorithm and tuning was performed by minimizing the integrated Brier score. The RSF model's output consisted of predicted survival probabilities for each subject over a 9-year period. The survival plots of patients who underwent TKR surgery at 1 year, 3 years, 5 years, and 7 years were provided in Fig. 1.

The statistical analyses, feature selection using the glmnet function within the Lasso Cox model, and time-to-TKR surgery prediction using the RSF model implemented through the autoSurv function (version 0.1.0), were performed using R Project for Statistical Computing Software (R version 4.3.1, R-Project.org). The DL models were trained in Python (version 3.10.4, Python Software Foundation, Wilmington, DE) and PyTorch

(version 1.11.0; pytorch.org). The GPU of Tesla V100−SXM2−32GB, compute capability: 7.0 was used for training the DL models.

**Feature selection**

All available radiograph image assessment measurements (31 quantitative, 19 semi-quantitative, and 1 alignment angle) and MRI image assessment measurements (92 quantitative, 88 semi-quantitative MOAKS) were utilized in this study. Due to variations in measurements obtained across different vendors, details of the image assessment measurements used in the study are provided in Table S2.

Central assessments of OA are based on three types of imaging modalities:

- Qualitative/Semi-quantitative: Presence/absence or ordinal scales for OA-related abnormalities (e.g., Kellgren-Lawrence grades, MOAKS score).
- Quantitative: Continuous measurements of OA-related features (e.g., cartilage volume, joint space width).
- Alignment: Dimensionless scores quantifying differences between individual knee shapes and a reference shape.

**Model prediction evaluation metrics**

Accuracy and the concordance index (C-index) were used as estimation evaluation metrics. Accuracy was calculated as:

$$ACC = 100 \times \frac{N_{\text{correct}}}{N_{\text{total}}}$$

where ACC: the accuracy of the TKR time prediction model,

$N_{\text{correct}}$: the number of patients whose predicted TKR time falls within ±1 year of the actual TKR time ($|y - \hat{y}| \leq 1$),

$N_{\text{total}}$: the total number of patients in the study.

The C-index, which measures the model's ability to rank survival times accurately based predicted risk of undergoing the TKR, was calculated as:

$$\text{C-index} = 100 \times \frac{\text{Concordant Pairs}}{\text{Total Pairs}}$$

where" Concordant Pairs" referred to the number of pairs where the patient with a shorter predicted time-to-TKR had the TKR surgery earlier than the patient with a longer predicted time-to-TKR and" Total Pairs" represented the total number of possible pairs where one patient's TKR time was known to be earlier or the same as the other patient's TKR time.

**Ablation Study**

The performance of utilizing clinical variables together with quantitative and semi-quantitative assessments from radiographs and MRI scans, DL features extracted from these imaging modalities, and their combination were analyzed using three different models: The Cox proportional hazards model, discrete-time generalized linear model, and RSF model. The experiments were conducted with the study cohort, and among them, the RSF model yielded the most favorable results.

**Survival Analysis**

Survival analysis tracks the occurrence of specific events (e.g., TKR) over time, plotting the likelihood (%) of remaining event-free up to a given follow-up period, termed as survival probability or cumulative survival, on the y-axis against the follow-up duration on the x-axis. Kaplan-Meier survival curves along with a graphical overview of the pertinent statistical parameters and commonly employed statistical methodologies for survival analysis were provided in Fig. 2 for all available radiographs in OAI database [2].

[2]S. H. Park, K. Han, and S. Y. Park, "Mistakes to avoid for accurate and transparent reporting of survival analysis in imaging research," Korean J. Radiol., vol. 22, no. 10, pp. 1587–1593, Oct. 2021

**Details of proposed model with best prediction performance**

The model with Lasso Cox feature selection, using clinical variables, and quantitative and semi-quantitative image assessments with self-supervised features from DESS and TSE MR scans and radiographs, achieved an accuracy of 75.2% and C-index of 85.3%. The confusion matrices for $\pm 1$year estimation ($|y - \hat{y}| \leq 1$) are shown in Fig. 3. Table S4 provides the features yielded the best time-to-TKR estimation accuracy of 75.2% and C-index of was calculated using the 'vimp' function from the 'randomForestSRC' package (version The importance 3.2.2) in R. To facilitate comparison, the scores were normalized to percentages. Higher percentages signified a greater influence on the model's predictions, implying that permuting the values of that variable had more effect on the model's predictive performance. Selected features and their relative importance ranked from highest to lowest scores were provided in Fig. 4. Additionally, the Cox proportional hazards model was fit to selected features and hazard ratios with 95% confidence intervals were calculated. The results were yielded in Table S5.

Table S1: Summary of study cohorts that underwent total knee replacement in the OAI, MOST, and internal datasets.

| Dataset | Imaging Type | Dataset Split | Number of Patients | Number of Images |
|---|---|---|---|---|
| OAI | MRI(TSE, DESS) | Train | 308 | 1572 |
| | | Validation | 41 | 246 |
| | | Test | 86 | 457 |
| | XRay | Train | 293 | 1551 |
| | | Validation | 41 | 253 |
| | | Test | 84 | 476 |
| MOST | MRI(TSE) | Test | 518 | 754 |
| | XRay | Test | 505 | 790 |
| Internal | XRay | Test | 164 | 164 |

Note: DESS: sagittal fat-suppressed three-dimensional dual-echo in steady state, Internal: internal hospital dataset, MOST= multi-center osteoarthritis study, No: number, OAI= osteoarthritis initiative, TSE: sagittal fat-suppressed intermediate- weighted turbo spin-echo.

Table S2: Details of image assessment measurements used in the study. In MRI quantitative readings, the vendors were matched for each patient. XX: visit prefix in month, 00: baseline, 01: 12 months, 03: 24 months, 05: 36 months, 06: 48 months, 08: 72 months, 10: 96 months.

| Imaging | Vendor | Measurements | Vendor | Dataset Name | Visits (Month) |
|---|---|---|---|---|---|
| XRay | Quantitative | Quantitative JSW | Duryea | kxr_qjsw_duryeaXX | 00,01,03,05,06,08,10 |
| | Semi-quantitative | K-L grade, IRFs | Boston Uni.-Felson, Aliabadi & Sack | kxr_sq_buXX | 00,01,03,05,06,08,10 |
| | Alignement | Femoral-tibial angle | Duryea | kxr fta duryeaXX | 00,01,03,05,06,08,10 |
| MRI | Quantitative | Quantitative cartilage | Eckstein | kMRI_QCart_EcksteinXX | 00,01,03,05,06 |
| | Semi-quantitative | MOAKS | BICL | kmri sq moaks biclXX | 00,01,03,05,06 |

Table S3: The AUC performances of using different models for prediction of time-to-TKR.

| † | DESS | TSE | XRay | Clinical | Measurements | Cox | GLM | RSF |
|---|---|---|---|---|---|---|---|---|
| | | | | ✓ | ✓ | 0.901 | 0.898 | 0.94 |
| | ✓ | ✓ | ✓ | | | 0.809 | 0.811 | 0.821 |
| | ✓ | ✓ | ✓ | ✓ | ✓ | 0.904 | 0.895 | 0.938 |
| ✓ | ✓ | ✓ | ✓ | ✓ | ✓ | 0.935 | 0.936 | 0.945 |

Note: Clinical: clinical variables, DESS: sagittal fat-suppressed three-dimensional dual-echo in steady state, GLM: generalized linear model, Measurements: quantitative and semi-quantitative image assessment measurements, RSF: random survival forest, TSE: sagittal fat-suppressed intermediate-weighted turbo spin-echo, †: LASSO Cox feature selection method was used before RSF model.

Table S4: 95 features selected by Lasso Cox applied to combined features (accuracy: 75.2%, C-index: 85.3%). Importance (%): the importance of each variable in the RSF model.

| Variables | | Importance(%) | Explanation of Variables |
|---|---|---|---|
| AGE | | 0.131 | Age |
| ALTMTH | | 0.065 | BL/FU kMRI reading (FE): mean cartilage thickness - lateral tibia (anterior) (aLT.ThCtAB) [mm] |
| AMTPD | | 0.006 | BL/FU kMRI reading (FE): % area of subchondral bone denuded of cartilage - medial tibia (anterior) (aMT.dAB%) [%] |
| ARTDOC | | 0.132 | Currently seeing doctor or other health care professional for arthritis |
| ARTDRCV | | 0.019 | Seeing doctor/other professional for knee arthritis (calc) |
| BISPHOS | | 0.05 | EV:Q58.Taken bisphosphonate medication (includes alendronate, risedronate...) to treat osteoporosis or Paget's disease, past 5 years |
| BLFCAAB | | 0.067 | BL/FU kMRI reading (FE): area of subchondral bone covered by cartilage - central lateral femur (cLF.cAB) |
| BMI | | 0.048 | BMI |
| BPARM | | 0.197 | Blod pressure |
| BPDAYCV | | 0.204 | How many days limit activities due to back pain, past 30 days (calc) |
| CBMFPD | | 0.024 | BL/FU kMRI reading (FE): % area of subchondral bone denuded of cartilage - central medial femur (center) (ccMF.dA%) [%] |
| CESD16 | | 0.06 | CES-D: how often enjoyed life, past week |
| CESD4 | | 0.094 | how often felt just as good as other people, past week |
| CHNFQCV | | 0.133 | Chondroitin sulfate frequency of use, past 6 months (calc) |
| CSTREP2 | | 0.094 | Repeated chair stands: trial 2 |
| DESS101 | | 0.08 | DL model features extracted from DESS MRI sequence |
| DESS170 | | 0.056 | DL model features extracted from DESS MRI sequence |
| DESS188 | | 0.016 | DL model features extracted from DESS MRI sequence |
| DESS38 | | 0.099 | DL model features extracted from DESS MRI sequence |
| DESS97 | | 0.084 | DL model features extracted from DESS MRI sequence |
| DILKN3 | | 0.189 | LEFT, knee difficulty: stand from sitting, last 7 days |
| DILKN7 | | 0.004 | LEFT knee difficulty: in car/out of car, last 7 days |
| EBLFPD | | 0.075 | BL/FU kMRI reading (FE): % area of subchondral bone denuded of cartilage - central lateral femur (external) (ecLF.dAB%) [%] |
| HLTHCAR | | 0.285 | Where usually go for health care or advice about health care |
| HLTHCOV | | 0.105 | Currently have any kind of health care coverage |
| HSMSS | | 0.078 | mental summary scale for the MOS 12-item short-form health survey (SF-12) v2 (calc) |
| HSPSS | | 0.014 | Physical summary scale for the MOS 12-item short-form health survey (SF-12) v2 (calc) |
| HYAINJR | | 0.046 | RIGHT knee hyaluronic acid injection, past 6 months (calc) |
| IBMFPD | | 0.028 | BL/FU kMRI reading (FE): % area of subchondral bone denuded of cartilage - central medial femur (internal) (icMF.dAB%) [%] |
| KEXAMK | | 0.1 | Knee exam: knee eligible to be examined (calc) |
| KNINJ | | 0.128 | EV: Q53.Either knee, injections for treatment of arthritis, past 6 months |
| KPMEDCV | | 0.097 | Either knee, used medication for pain, aching or stiffness more than half the days of a month, past 12 months (calc, used for study eligibility) |
| KQOL1 | | 0.02 | Quality of life: how often aware of problems with knee(s) |
| KQOL4 | | 0.12 | Quality of life: in general, how much difficulty have with knee(s) |
| KSXRKN4 | | 0.074 | RIGHT knee symptoms: straighten knee fully, last 7 days |
| LKABPN | | 0.29 | LEFT knee exam: anserine bursa, pain/tenderness present on exam |
| LTPMEBE | | 0.273 | BL/FU kXR reading (JD): lateral tibial plateau margin is the same as the bone edge |
| MACLTR | | 0.323 | BL/FU kMRI reading (BI): MOAKS: ACL tear |
| MBMNFMA | | 0.073 | BL/FU kMRI reading (BI): MOAKS: number of BML lesions - femur medial anterior (trochlear) |
| MBMPFMA | | 0.184 | MOAKS: BML (% lesion that is edema) - femur medial anterior (trochlear) |
| MBMPPL | | 0.122 | BL/FU kMRI reading (BI): MOAKS: BML (% lesion that is edema) - patella lateral |
| MBMPPM | | 0.105 | BL/FU kMRI reading (BI): MOAKS: BML (% lesion that is edema) - patella medial |
| MBMPSS | | 0.157 | MOAKS: BML (% lesion that is edema) - tibia sub-spinous |
| MBMSTLA | | 0.097 | BL/FU kMRI reading (BI): MOAKS: BML size - tibia lateral anterior |
| MBMSTLP | | 0.079 | BL/FU kMRI reading (BI): MOAKS: BML size - tibia lateral posterior |
| MCMFLC | | 0.083 | BL/FU kMRI reading (BI): MOAKS: cartilage morphology - femur lateral central |
| MCMFMC | | 0.187 | BL/FU kMRI reading (BI): MOAKS: cartilage morphology - femur medial central |
| MCMPL | | 0.046 | BL/FU kMRI reading (BI): MOAKS: cartilage morphology - patella lateral |
| MCMTLA | | 0.046 | BL/FU kMRI reading (BI): MOAKS: cartilage morphology - tibia lateral anterior |
| MCMTMA | | 0.129 | BL/FU kMRI reading (BI): MOAKS: cartilage morphology - tibia medial anterior |
| MCMTMC | | 0.091 | BL/FU kMRI reading (BI): MOAKS: cartilage morphology - tibia medial central |
| MEDINS | | 0.063 | Have any health insurance plan that pays for all or part of cost of prescription medicines |
| MMXLL | | 0.135 | BL/FU kMRI reading (BI): MOAKS: lateral meniscal extrusion - laterally |
| OTHVP1 | | 0.111 | EV:Phlebotomy: traumatic venipuncture, other (first draw) (calc) |
| PASE6 | | 0.092 | Leisure activities: muscle strength/endurance, past 7 days |
| PNMEDT | | 0.098 | Take any pain medication today (include both prescription and over-the-counter medications for any type of pain) |
| RELA12 | | 0.236 | do relaxation or mind-body activities, such as meditation, deep breathing or visualization, for arthritis or joint pain, past 12 months |
| RKABPN | | 0.332 | RIGHT knee exam: anserine bursa, pain/tenderness present on exam |
| RXASPRN | | 0.112 | Rx Aspirin use indicator (calc) |
| RXBISPH | | 0.135 | Bisphosphonate use indicator (calc) |
| SF7 | | 0.127 | how often emotional problems result in not doing work or activities as carefully as usual, past 4 weeks |
| SF8 | | 0.102 | how much did pain interfere with normal work (include work outside home and housework), past 4 weeks |
| STINJCV | | 0.027 | Either knee, steroid injection (cortisone/corticosteroid), past 6 months (calc) |
| STRINJL | | 0.041 | LEFT knee steroid injection, past 6 months (calc) |
| TSE106 | | 0.125 | DL model features extracted from TSE MRI sequence |
| TSE123 | | 0.041 | DL model features extracted from TSE MRI sequence |
| TSE200 | | 0.008 | DL model features extracted from TSE MRI sequence |
| TSE246 | | 0.013 | DL model features extracted from TSE MRI sequence |
| TSE48 | | 0.175 | DL model features extracted from TSE MRI sequence |
| TSE74 | | 0.033 | DL model features extracted from TSE MRI sequence |
| VITM12 | | 0.367 | Use vitamins or minerals, such as selenium or vitamin C or D, for arthritis or joint pain, past 12 months |
| WMTCTS | | 0.375 | SD of cartilage thickness - medial tibia |
| WPRKN2 | | 0.093 | RIGHT knee pain: stairs, last 7 days |
| XRay100 | | 0.113 | DL model features extracted from radiographs |
| XRay119 | | 0.094 | DL model features extracted from radiographs |
| XRay17 | | 0.107 | DL model features extracted from radiographs |
| XRay202 | | 0.112 | DL model features extracted from radiographs |
| XRay251 | | 0.078 | DL model features extracted from radiographs |
| XRay263 | | 0.027 | DL model features extracted from radiographs |
| XRay279 | | 0.077 | DL model features extracted from radiographs |
| XRay291 | | 0.07 | DL model features extracted from radiographs |
| XRay310 | | 0.209 | DL model features extracted from radiographs |
| XRay312 | | 0.069 | DL model features extracted from radiographs |
| XRay340 | | 0.094 | DL model features extracted from radiographs |
| XRay341 | | 0.033 | DL model features extracted from radiographs |
| XRay376 | | 0.016 | DL model features extracted from radiographs |
| XRay378 | | 0.087 | DL model features extracted from radiographs |
| XRay401 | | 0.055 | DL model features extracted from radiographs |
| XRay450 | | 0.046 | DL model features extracted from radiographs |
| XRay459 | | 0.002 | DL model features extracted from radiographs |
| XRay483 | | 0.189 | DL model features extracted from radiographs |
| XRay53 | | 0.165 | DL model features extracted from radiographs |
| XRJSL | | 0.016 | BL/FU kXR reading (BU): joint space narrowing (OARSI grades 0-3) lateral compartment |
| XRJSM | | 0.062 | BL/FU kXR reading (BU): joint space narrowing (OARSI grades 0-3) medial compartment |
| XRKL | | 0.096 | BL/FU kXR reading (BU): Kellgren and Lawrence (grades 0-4) |

Table S5: Cox proportional hazard model. Table S5 continued on the next page.

| **Variables** | **Cox Proportional Hazard (PH) Model** | | | | | |
|---|---|---|---|---|---|---|
| | coef | exp(coef) | se(coef) | z | Pr(>\|z\|) | |
| CBMFPD | -0.024 | 0.977 | 0.077 | -0.303 | 0.762 | |
| WMTCTS | 0.375 | 1.456 | 0.064 | 5.905 | 0 | *** |
| EBLFPD | 0.075 | 1.077 | 0.07 | 1.072 | 0.284 | |
| AMTPD | -0.006 | 0.994 | 0.047 | -0.13 | 0.896 | |
| BLFCAAB | 0.067 | 1.069 | 0.065 | 1.031 | 0.303 | |
| IBMFPD | 0.028 | 1.028 | 0.048 | 0.579 | 0.563 | |
| ALTMTH | 0.065 | 1.067 | 0.066 | 0.975 | 0.33 | |
| MCMPL | 0.046 | 1.048 | 0.058 | 0.808 | 0.419 | |
| MCMFMC | 0.187 | 1.205 | 0.073 | 2.552 | 0.011 | * |
| MCMFLC | 0.083 | 1.087 | 0.065 | 1.282 | 0.2 | |
| MCMTMA | 0.129 | 1.137 | 0.078 | 1.649 | 0.099 | . |
| MCMTLA | 0.046 | 1.048 | 0.056 | 0.822 | 0.411 | |
| MCMTMC | 0.091 | 1.095 | 0.078 | 1.16 | 0.246 | |
| MBMSTLA | 0.097 | 1.102 | 0.043 | 2.248 | 0.025 | * |
| MBMSTLP | 0.079 | 1.082 | 0.044 | 1.793 | 0.073 | . |
| MBMPFMA | -0.184 | 0.832 | 0.11 | -1.68 | 0.093 | . |
| MBMPSS | 0.157 | 1.17 | 0.057 | 2.754 | 0.006 | ** |
| MBMPPM | -0.105 | 0.9 | 0.06 | -1.747 | 0.081 | . |
| MBMPPL | -0.122 | 0.885 | 0.064 | -1.902 | 0.057 | . |
| MBMNFMA | 0.073 | 1.076 | 0.159 | 0.46 | 0.645 | |
| MMXLL | 0.135 | 1.144 | 0.071 | 1.907 | 0.056 | . |
| MACLTR | -0.323 | 0.724 | 0.078 | -4.149 | 0 | *** |
| TSE48 | 0.175 | 1.191 | 0.066 | 2.631 | 0.009 | ** |
| TSE74 | -0.033 | 0.967 | 0.108 | -0.31 | 0.756 | |
| TSE106 | -0.125 | 0.882 | 0.074 | -1.684 | 0.092 | . |
| TSE123 | 0.041 | 1.042 | 0.086 | 0.482 | 0.63 | |
| TSE200 | -0.008 | 0.992 | 0.064 | -0.119 | 0.906 | |
| TSE246 | -0.013 | 0.988 | 0.052 | -0.242 | 0.809 | |
| OTHVP1 | 0.111 | 1.118 | 0.039 | 2.828 | 0.005 | ** |
| SF7 | 0.127 | 1.135 | 0.062 | 2.058 | 0.04 | * |
| SF8 | 0.102 | 1.107 | 0.088 | 1.159 | 0.246 | |
| WPRKN2 | 0.093 | 1.098 | 0.056 | 1.661 | 0.097 | . |
| KSXRKN4 | 0.074 | 1.077 | 0.043 | 1.701 | 0.089 | . |
| DILKN3 | 0.189 | 1.208 | 0.072 | 2.636 | 0.008 | ** |
| DILKN7 | -0.004 | 0.996 | 0.071 | -0.061 | 0.951 | |
| KQOL1 | -0.02 | 0.981 | 0.069 | -0.284 | 0.776 | |
| KQOL4 | 0.12 | 1.128 | 0.067 | 1.794 | 0.073 | . |
| HSPSS | 0.014 | 1.014 | 0.093 | 0.152 | 0.879 | |
| HSMSS | 0.078 | 1.082 | 0.065 | 1.204 | 0.229 | |

Note: For Lasso Cox model, hazard ratios (exp(coef)) were calculated as the exponential of Lasso Cox coefficients of the selected features. Optimal lambda value selected by cross-validation was 0.03677. For Cox PH model, a Cox proportional hazards model was fit to features selected using the Lasso Cox model. Hazard ratios (exp(coef)) were calculated as the exponential of Cox coefficients of the selected features. Concordance=0.876 (se=0.005), likelihood ratio test=1476 , Wald test=1056, and score (logrank) test=1743 on 95 df, $p \leq 2e-16$. Significant codes: '***' 0.001, '**' 0.01, '*' 0.05, '.' 0.1, ' ' 1.

Table S5: Continued.

| Variables | Cox Proportional Hazard (PH) Model | | | | | |
|---|---|---|---|---|---|---|
| | coef | exp(coef) | se(coef) | z | Pr(>\|z\|) | |
| BPDAYCV | -0.204 | 0.815 | 0.047 | -4.357 | 0 | *** |
| ARTDOC | 0.132 | 1.141 | 0.078 | 1.689 | 0.091 | . |
| CESD4 | 0.094 | 1.099 | 0.048 | 1.988 | 0.047 | * |
| CESD16 | 0.06 | 1.062 | 0.053 | 1.147 | 0.251 | |
| PNMEDT | 0.098 | 1.103 | 0.044 | 2.238 | 0.025 | * |
| ARTDRCV | 0.019 | 1.02 | 0.08 | 0.245 | 0.807 | |
| HYAINJR | 0.046 | 1.047 | 0.038 | 1.189 | 0.234 | |
| STRINJL | 0.041 | 1.042 | 0.064 | 0.642 | 0.521 | |
| CHNFQCV | 0.133 | 1.142 | 0.044 | 3.01 | 0.003 | ** |
| STINJCV | 0.027 | 1.027 | 0.076 | 0.355 | 0.723 | |
| RXBISPH | 0.135 | 1.144 | 0.078 | 1.73 | 0.084 | . |
| RXASPRN | -0.112 | 0.894 | 0.057 | -1.945 | 0.052 | . |
| BPARM | -0.197 | 0.821 | 0.056 | -3.515 | 0 | *** |
| CSTREP2 | 0.094 | 1.098 | 0.044 | 2.137 | 0.033 | * |
| RKABPN | 0.332 | 1.394 | 0.069 | 4.79 | 0 | *** |
| LKABPN | 0.29 | 1.336 | 0.07 | 4.135 | 0 | *** |
| KEXAMK | -0.1 | 0.905 | 0.046 | -2.17 | 0.03 | * |
| HLTHCAR | -0.285 | 0.752 | 0.067 | -4.272 | 0 | *** |
| HLTHCOV | 0.105 | 1.111 | 0.075 | 1.408 | 0.159 | |
| MEDINS | 0.063 | 1.065 | 0.055 | 1.143 | 0.253 | |
| PASE6 | 0.092 | 1.096 | 0.042 | 2.163 | 0.031 | * |
| AGE | 0.131 | 1.139 | 0.057 | 2.298 | 0.022 | * |
| XRJSM | 0.062 | 1.064 | 0.081 | 0.77 | 0.441 | |
| XRJSL | 0.016 | 1.016 | 0.082 | 0.198 | 0.843 | |
| BMI | 0.096 | 1.101 | 0.074 | 1.307 | 0.191 | |
| XRKL | -0.048 | 0.953 | 0.081 | -0.592 | 0.554 | |
| LTPMEBE | 0.273 | 1.314 | 0.071 | 3.819 | 0 | *** |
| DESS38 | 0.099 | 1.104 | 0.045 | 2.181 | 0.029 | * |
| DESS97 | 0.084 | 1.088 | 0.047 | 1.783 | 0.075 | . |
| DESS101 | -0.08 | 0.923 | 0.051 | -1.576 | 0.115 | |
| DESS170 | 0.056 | 1.058 | 0.046 | 1.23 | 0.219 | |
| DESS188 | 0.016 | 1.016 | 0.051 | 0.314 | 0.754 | |
| XRay17 | -0.107 | 0.899 | 0.051 | -2.099 | 0.036 | * |
| XRay53 | 0.165 | 1.18 | 0.078 | 2.114 | 0.035 | * |
| XRay100 | 0.113 | 1.119 | 0.047 | 2.418 | 0.016 | * |
| XRay119 | -0.094 | 0.91 | 0.051 | -1.862 | 0.063 | . |
| XRay202 | 0.112 | 1.119 | 0.062 | 1.81 | 0.07 | . |
| XRay251 | 0.078 | 1.081 | 0.053 | 1.472 | 0.141 | |
| XRay263 | -0.027 | 0.974 | 0.064 | -0.421 | 0.674 | |
| XRay279 | 0.077 | 1.08 | 0.054 | 1.434 | 0.152 | |
| XRay291 | 0.07 | 1.072 | 0.058 | 1.215 | 0.224 | |
| XRay310 | -0.209 | 0.812 | 0.058 | -3.602 | 0 | *** |
| XRay312 | 0.069 | 1.072 | 0.053 | 1.306 | 0.191 | |
| XRay340 | 0.094 | 1.099 | 0.05 | 1.875 | 0.061 | . |
| XRay341 | 0.033 | 1.034 | 0.056 | 0.594 | 0.552 | |
| XRay376 | 0.016 | 1.016 | 0.066 | 0.241 | 0.809 | |
| XRay378 | -0.087 | 0.916 | 0.07 | -1.25 | 0.211 | |
| XRay401 | -0.055 | 0.946 | 0.053 | -1.037 | 0.3 | |
| XRay450 | 0.046 | 1.047 | 0.067 | 0.687 | 0.492 | |
| XRay459 | -0.002 | 0.998 | 0.058 | -0.036 | 0.971 | |
| XRay483 | -0.189 | 0.828 | 0.052 | -3.61 | 0 | *** |

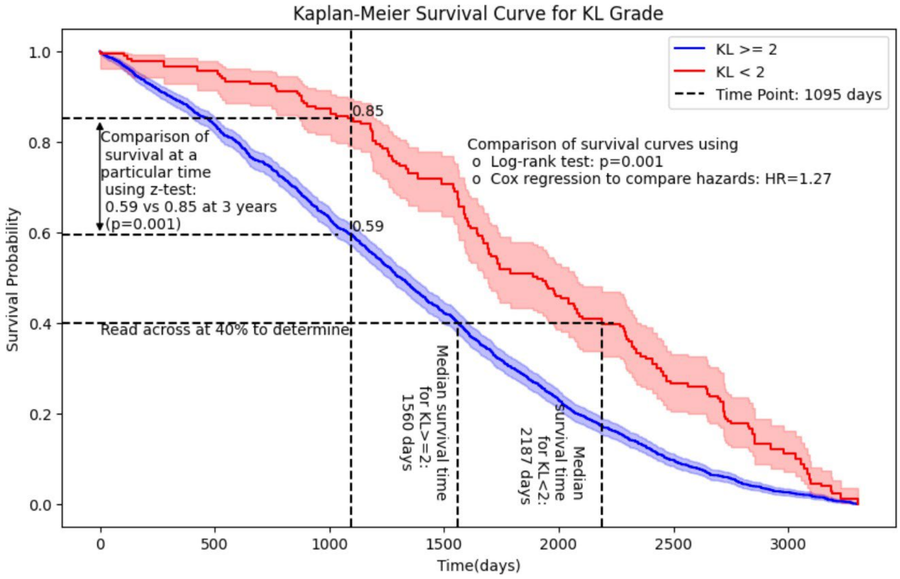


Figure S1: The Kaplan-Meier survival curves. The hazard ratio of 1.27 indicates that the risk of undergoing TKR is 1.27 times greater in the patients with the $KL \geq 2$ compared to those $KL < 2$. Kellgren Lawrance (KL) grade $\geq 2$ defines the radiographic knee OA in KL system.

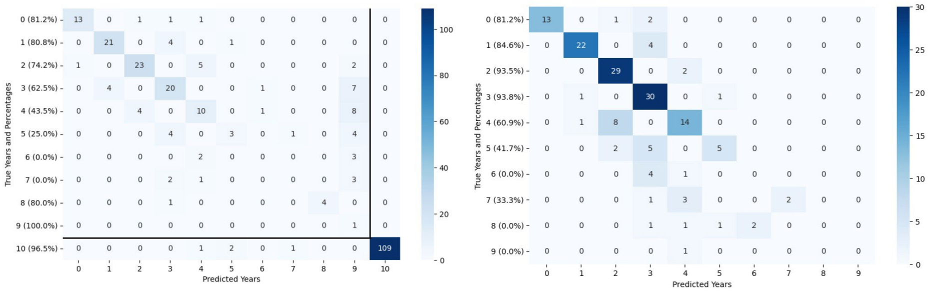


(a) (b)

Figure S2: Confusion matrices for ±1 year estimation ($|y - \hat{y}| \leq 1$) for the following models

a) †(Self-supervised (DESS+TSE)+Pretrained-ResNet(XRay)+Clinical+Measurements) with data including both the subjects underwent TKR in 9-years and right-censored controls),

b) †(Supervised (DESS+TSE+XRay)+Clinical+Measurements) with only the subjects underwent TKR in 9-years.†: LASSO Cox feature selection method was used before RSF model.

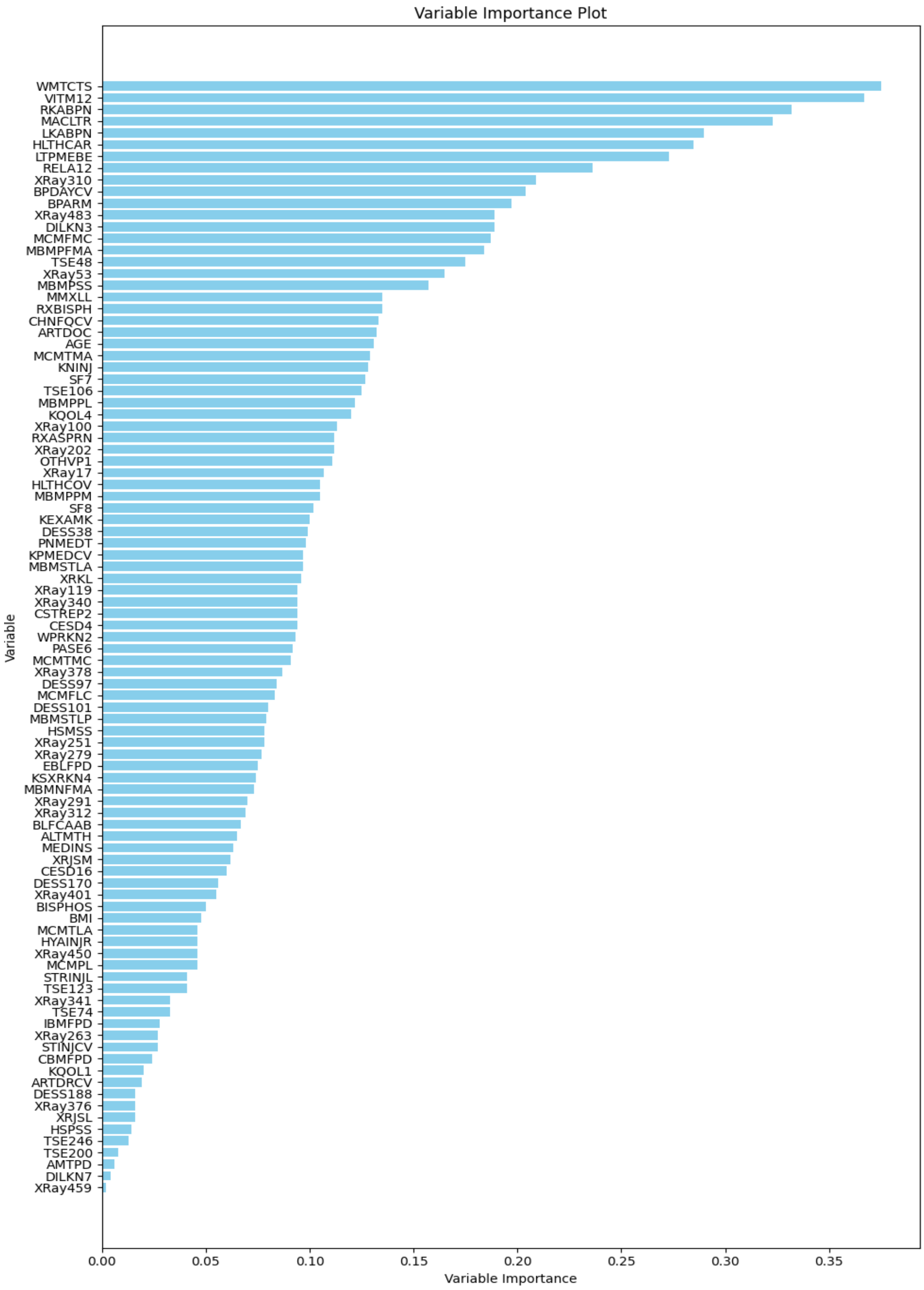


Figure S3: Ranked importance of selected features in random survival model (accuracy: 75.2%, C-index: 85.3%).

**File Names**

**OAI Database:** The ID, follow-up information, and knee side are provided below. In the file names, 'm' represents months; for example, '12m' indicates a follow-up of 12 months.

9000798_00m_LEFT, 9001695_00m_RIGHT, 9001897_00m_LEFT, 9001897_00m_RIGHT, 9002116_00m_LEFT, 9002430_12m_RIGHT, 9002430_24m_RIGHT, 9002430_36m_RIGHT, 9002430_48m_RIGHT, 9002817_00m_RIGHT, 9003316_00m_RIGHT, 9003380_00m_RIGHT, 9003895_00m_RIGHT, 9004175_00m_RIGHT, 9004905_00m_RIGHT, 9005132_00m_LEFT, 9005132_12m_LEFT, 9005132_24m_LEFT, 9006407_00m_RIGHT, 9006723_00m_LEFT, 9009927_00m_RIGHT, 9010370_00m_RIGHT, 9011918_12m_LEFT, 9013798_00m_LEFT, 9014209_00m_RIGHT, 9014797_00m_RIGHT, 9014883_00m_LEFT, 9014883_12m_LEFT, 9014883_24m_LEFT, 9014883_48m_LEFT, 9015363_00m_RIGHT, 9015363_12m_RIGHT, 9015363_24m_RIGHT, 9015363_36m_RIGHT, 9015718_00m_LEFT, 9015798_00m_RIGHT, 9016304_00m_RIGHT, 9016403_00m_RIGHT, 9021102_00m_LEFT, 9021428_00m_RIGHT, 9022789_00m_RIGHT, 9022902_00m_LEFT, 9024940_00m_RIGHT, 9025191_00m_RIGHT, 9026416_00m_LEFT, 9027422_00m_LEFT, 9027422_12m_LEFT, 9027422_24m_LEFT, 9028418_00m_RIGHT, 9028418_12m_RIGHT, 9028418_24m_RIGHT, 9028418_36m_RIGHT, 9028786_00m_LEFT, 9029913_00m_RIGHT, 9030718_00m_LEFT, 9030718_00m_RIGHT, 9030718_12m_LEFT, 9030718_12m_RIGHT, 9030718_24m_RIGHT, 9030718_36m_RIGHT, 9031141_00m_RIGHT, 9031426_00m_RIGHT, 9031426_12m_RIGHT, 9033937_00m_RIGHT, 9034451_00m_LEFT, 9034451_00m_RIGHT, 9034451_12m_LEFT, 9034451_12m_RIGHT, 9034451_24m_LEFT, 9034451_24m_RIGHT, 9034451_36m_RIGHT, 9034451_48m_RIGHT, 9035449_00m_RIGHT, 9037494_00m_RIGHT, 9037823_00m_RIGHT, 9040390_00m_RIGHT, 9040944_00m_RIGHT, 9040944_12m_RIGHT, 9040944_24m_RIGHT, 9040944_36m_RIGHT, 9040944_48m_RIGHT, 9043005_00m_LEFT, 9043507_00m_RIGHT, 9048192_00m_RIGHT, 9048789_00m_LEFT, 9048789_24m_LEFT, 9048789_48m_LEFT, 9048898_00m_LEFT, 9049007_00m_LEFT, 9049185_00m_RIGHT, 9049223_00m_LEFT, 9049447_00m_RIGHT, 9049479_00m_LEFT, 9049479_00m_RIGHT, 9049507_00m_LEFT, 9049706_00m_LEFT, 9053047_00m_LEFT, 9053047_24m_RIGHT, 9053047_36m_RIGHT, 9054370_00m_LEFT, 9054866_00m_LEFT, 9055038_00m_LEFT, 9055836_00m_RIGHT, 9057150_00m_LEFT, 9057150_12m_LEFT, 9058146_00m_LEFT, 9058692_00m_RIGHT, 9059391_00m_RIGHT, 9064464_00m_RIGHT, 9064927_00m_LEFT, 9064927_00m_RIGHT, 9065272_00m_RIGHT, 9065952_00m_LEFT, 9066155_00m_RIGHT, 9066155_12m_RIGHT, 9066155_24m_RIGHT, 9066425_00m_RIGHT, 9067425_00m_LEFT, 9068894_00m_RIGHT, 9069117_00m_RIGHT, 9069393_00m_LEFT, 9070207_00m_LEFT, 9070207_00m_RIGHT, 9070903_00m_RIGHT, 9071463_00m_LEFT, 9071669_00m_RIGHT, 9071924_00m_LEFT, 9074878_00m_LEFT, 9074878_00m_RIGHT, 9074878_12m_LEFT, 9074878_12m_RIGHT, 9074878_24m_LEFT, 9074878_24m_RIGHT, 9074878_36m_LEFT, 9075815_00m_LEFT, 9075815_00m_RIGHT, 9075900_00m_LEFT, 9075939_00m_RIGHT, 9076879_00m_RIGHT, 9082159_00m_RIGHT, 9085290_00m_LEFT, 9085290_12m_LEFT, 9085290_12m_RIGHT, 9085290_24m_LEFT, 9085290_36m_LEFT, 9086133_00m_LEFT, 9086204_00m_RIGHT, 9086204_12m_RIGHT, 9086204_24m_RIGHT, 9086868_00m_LEFT, 9087632_00m_LEFT, 9087632_12m_LEFT, 9087632_24m_LEFT, 9087632_48m_LEFT, 9088414_00m_LEFT, 9089119_00m_LEFT, 9093108_00m_LEFT, 9093584_00m_RIGHT, 9095715_00m_RIGHT, 9095865_00m_LEFT, 9095865_12m_LEFT, 9095865_24m_LEFT,

9096724_00m_LEFT, 9096724_12m_LEFT, 9096724_24m_LEFT, 9098409_00m_LEFT, 9098882_00m_RIGHT, 9098924_00m_LEFT, 9099360_00m_RIGHT, 9099360_12m_RIGHT, 9099363_00m_LEFT, 9100862_00m_LEFT, 9101854_00m_LEFT, 9101854_00m_RIGHT, 9103783_00m_LEFT, 9103783_12m_LEFT, 9107980_00m_RIGHT, 9107980_12m_RIGHT, 9107980_24m_RIGHT, 9109448_00m_RIGHT, 9112020_00m_LEFT, 9112495_00m_RIGHT, 9112976_00m_RIGHT, 9115049_00m_LEFT, 9115049_12m_LEFT, 9115049_36m_LEFT, 9115049_48m_LEFT, 9117066_00m_LEFT, 9117066_00m_RIGHT, 9117066_12m_LEFT, 9117066_12m_RIGHT, 9117066_24m_LEFT, 9117066_24m_RIGHT, 9117066_36m_LEFT, 9117066_36m_RIGHT, 9117066_48m_LEFT, 9117066_48m_RIGHT, 9118061_00m_LEFT, 9118430_00m_RIGHT, 9118689_00m_LEFT, 9120059_00m_RIGHT, 9121285_00m_LEFT, 9122517_00m_RIGHT, 9122877_00m_RIGHT, 9126014_00m_LEFT, 9126260_00m_LEFT, 9127180_00m_LEFT, 9127180_12m_LEFT, 9127180_24m_LEFT, 9128270_00m_LEFT, 9128270_12m_LEFT, 9128270_24m_LEFT, 9128270_36m_LEFT, 9129226_00m_RIGHT, 9129270_00m_RIGHT, 9131969_00m_RIGHT, 9133464_00m_RIGHT, 9134955_00m_LEFT, 9135342_00m_RIGHT, 9135838_00m_RIGHT, 9135838_12m_RIGHT, 9137248_00m_LEFT, 9137556_00m_RIGHT, 9140556_00m_RIGHT, 9142896_00m_RIGHT, 9144057_00m_RIGHT, 9144314_00m_RIGHT, 9144384_00m_RIGHT, 9145695_00m_RIGHT, 9145709_00m_RIGHT, 9146709_00m_RIGHT, 9148828_00m_LEFT, 9148828_00m_RIGHT, 9149367_00m_RIGHT, 9150876_00m_LEFT, 9154699_00m_LEFT, 9154699_12m_LEFT, 9154699_24m_LEFT, 9154793_00m_LEFT, 9157384_00m_LEFT, 9157384_00m_RIGHT, 9158391_00m_LEFT, 9158391_00m_RIGHT, 9158391_12m_LEFT, 9158391_12m_RIGHT, 9158391_24m_LEFT, 9158391_24m_RIGHT, 9158391_36m_RIGHT, 9158391_48m_RIGHT, 9159401_00m_LEFT, 9159401_12m_LEFT, 9159401_24m_LEFT, 9159804_00m_LEFT, 9160801_00m_RIGHT, 9160801_12m_RIGHT, 9161024_00m_RIGHT, 9167541_00m_LEFT, 9168534_00m_RIGHT, 9168770_00m_RIGHT, 9168770_12m_RIGHT, 9168770_24m_RIGHT, 9170920_00m_RIGHT, 9171580_00m_RIGHT, 9171766_00m_LEFT, 9173792_00m_RIGHT, 9173792_12m_RIGHT, 9174216_00m_LEFT, 9174216_00m_RIGHT, 9174216_12m_LEFT, 9174216_12m_RIGHT, 9174216_24m_LEFT, 9174216_24m_RIGHT, 9174216_48m_LEFT, 9175691_00m_LEFT, 9176992_00m_RIGHT, 9177091_00m_LEFT, 9177408_00m_RIGHT, 9177408_12m_RIGHT, 9177408_24m_RIGHT, 9178780_00m_RIGHT, 9179789_00m_RIGHT, 9179789_12m_RIGHT, 9179789_24m_RIGHT, 9182051_00m_LEFT, 9184556_00m_RIGHT, 9184790_00m_RIGHT, 9187064_00m_RIGHT, 9187064_24m_RIGHT, 9188345_00m_RIGHT, 9192540_00m_LEFT, 9193863_00m_RIGHT, 9194300_00m_LEFT, 9194300_00m_RIGHT, 9194300_12m_LEFT, 9194300_12m_RIGHT, 9194860_00m_RIGHT, 9196959_00m_LEFT, 9197466_00m_RIGHT, 9198531_00m_LEFT, 9199024_00m_RIGHT, 9201046_00m_RIGHT, 9201046_24m_RIGHT, 9201046_36m_RIGHT, 9201046_48m_RIGHT, 9202456_00m_LEFT, 9204055_00m_RIGHT, 9206450_00m_LEFT, 9207458_00m_LEFT, 9208016_00m_RIGHT, 9208400_00m_RIGHT, 9208400_12m_RIGHT, 9210045_00m_LEFT, 9210505_00m_RIGHT, 9212052_00m_RIGHT, 9212451_00m_RIGHT, 9212530_00m_LEFT, 9214480_00m_LEFT, 9214480_00m_RIGHT, 9215390_00m_RIGHT, 9215922_00m_RIGHT, 9216735_00m_RIGHT, 9217260_00m_LEFT, 9217481_00m_RIGHT, 9217512_00m_RIGHT, 9218916_00m_LEFT, 9218916_12m_LEFT, 9218935_00m_RIGHT, 9218935_12m_RIGHT, 9218935_24m_RIGHT, 9218993_00m_LEFT, 9223040_00m_LEFT, 9223980_00m_LEFT, 9223980_00m_RIGHT, 9225063_00m_LEFT, 9225592_00m_LEFT, 9226874_00m_RIGHT, 9230073_00m_RIGHT, 9230073_12m_RIGHT, 9232078_00m_LEFT, 9233578_00m_RIGHT, 9234555_00m_LEFT, 9235666_00m_LEFT, 9235666_12m_LEFT, 9235666_12m_RIGHT, 9235666_24m_LEFT, 9235666_24m_RIGHT, 9238970_00m_LEFT, 9239552_00m_RIGHT,

9241014_00m_RIGHT, 9241544_00m_LEFT, 9241544_00m_RIGHT, 9245760_00m_RIGHT, 9248131_00m_LEFT, 9248131_12m_LEFT,

9248131_24m_LEFT, 9250401_00m_LEFT, 9250756_00m_RIGHT, 9251077_00m_RIGHT, 9251736_00m_LEFT, 9252019_00m_RIGHT,

9252130_00m_RIGHT, 9252576_00m_RIGHT, 9252629_00m_LEFT, 9252748_00m_RIGHT, 9255429_00m_LEFT, 9255535_00m_RIGHT,

9255567_00m_RIGHT, 9255884_00m_LEFT, 9256567_00m_RIGHT, 9256759_00m_RIGHT, 9258562_00m_LEFT, 9258562_00m_RIGHT,

9258562_12m_LEFT, 9258562_12m_RIGHT, 9258562_24m_LEFT, 9258562_24m_RIGHT, 9258562_36m_LEFT, 9258562_48m_LEFT,

9258864_00m_LEFT, 9259866_00m_LEFT, 9259866_12m_LEFT, 9260058_00m_RIGHT, 9261372_00m_LEFT, 9262506_00m_LEFT,

9263504_00m_RIGHT, 9263504_12m_RIGHT, 9263504_24m_RIGHT, 9263504_36m_RIGHT, 9264046_00m_LEFT, 9264046_00m_RIGHT,

9266027_00m_RIGHT, 9266274_00m_LEFT, 9267366_00m_RIGHT, 9267648_00m_RIGHT, 9267719_00m_RIGHT, 9268652_00m_RIGHT,

9269435_00m_RIGHT, 9269510_00m_RIGHT, 9270026_00m_RIGHT, 9272198_00m_LEFT, 9273128_00m_RIGHT, 9273128_12m_RIGHT,

9273128_24m_RIGHT, 9273128_36m_RIGHT, 9273128_48m_RIGHT, 9275258_00m_RIGHT, 9275309_00m_RIGHT, 9276150_00m_RIGHT,

9276849_00m_RIGHT, 9277154_00m_RIGHT, 9279291_00m_RIGHT, 9279291_12m_RIGHT, 9279291_24m_RIGHT, 9279291_36m_RIGHT,

9279291_48m_RIGHT, 9280204_00m_RIGHT, 9280249_00m_LEFT, 9281187_00m_LEFT, 9281187_12m_LEFT, 9282203_00m_RIGHT,

9282257_00m_RIGHT, 9282330_00m_RIGHT, 9283903_00m_RIGHT, 9284505_00m_RIGHT, 9284795_00m_RIGHT, 9285212_00m_LEFT,

9285212_12m_LEFT, 9285212_24m_LEFT, 9287346_00m_RIGHT, 9288025_00m_RIGHT, 9289212_00m_LEFT, 9290697_00m_LEFT,

9291078_00m_RIGHT, 9291078_12m_RIGHT, 9291078_24m_RIGHT, 9291078_36m_RIGHT, 9291206_00m_RIGHT, 9292081_00m_LEFT,

9292234_00m_RIGHT, 9294761_00m_LEFT, 9294761_12m_LEFT, 9294761_24m_LEFT, 9295400_00m_RIGHT, 9297051_00m_LEFT,

9297051_00m_RIGHT, 9297511_00m_LEFT, 9298541_00m_LEFT, 9298541_12m_LEFT, 9298541_24m_LEFT, 9299531_00m_LEFT,

9300338_00m_LEFT, 9300338_00m_RIGHT, 9301332_00m_RIGHT, 9303539_00m_LEFT, 9304828_00m_LEFT, 9305336_00m_LEFT,

9305487_00m_RIGHT, 9308099_00m_RIGHT, 9310332_00m_RIGHT, 9310472_00m_LEFT, 9311154_00m_LEFT, 9311154_12m_LEFT,

9311154_24m_LEFT, 9313233_00m_RIGHT, 9313233_12m_RIGHT, 9313330_00m_RIGHT, 9314101_12m_RIGHT, 9314305_00m_LEFT,

9314340_00m_RIGHT, 9315555_00m_LEFT, 9316108_00m_LEFT, 9316359_00m_RIGHT, 9316905_00m_LEFT, 9316905_00m_RIGHT,

9316905_12m_LEFT, 9316905_24m_LEFT, 9316905_36m_LEFT, 9316905_48m_LEFT, 9318369_00m_RIGHT, 9318527_00m_LEFT,

9319367_00m_RIGHT, 9321380_00m_RIGHT, 9322109_00m_LEFT, 9322109_00m_RIGHT, 9322109_12m_LEFT, 9322109_12m_RIGHT,

9322109_24m_LEFT, 9322109_24m_RIGHT, 9322109_36m_RIGHT, 9322401_00m_RIGHT, 9323849_00m_LEFT, 9324400_00m_RIGHT,

9326076_00m_RIGHT, 9327385_00m_RIGHT, 9328332_00m_RIGHT, 9328584_00m_LEFT, 9330147_00m_RIGHT, 9330155_00m_RIGHT,

9331053_00m_RIGHT, 9331465_00m_RIGHT, 9331465_24m_RIGHT, 9331465_48m_RIGHT, 9332345_00m_LEFT, 9332345_00m_RIGHT,

9332345_12m_LEFT, 9332345_12m_RIGHT, 9332345_24m_LEFT, 9332345_24m_RIGHT, 9332345_36m_LEFT, 9332345_36m_RIGHT,

9332414_00m_RIGHT, 9332791_00m_RIGHT, 9335072_00m_LEFT, 9335072_12m_LEFT, 9335072_24m_LEFT, 9335358_00m_RIGHT,

9336300_00m_LEFT, 9336300_12m_LEFT, 9336300_24m_LEFT, 9338059_00m_LEFT, 9338479_00m_RIGHT, 9339252_00m_LEFT,

9340139_00m_RIGHT, 9340139_12m_RIGHT, 9340139_24m_RIGHT, 9340283_00m_RIGHT, 9340283_12m_RIGHT, 9340283_24m_RIGHT,

9340283_36m_RIGHT, 9340335_00m_RIGHT, 9341240_00m_LEFT, 9341240_00m_RIGHT, 9341240_12m_LEFT, 9341240_24m_LEFT,

9341240_48m_LEFT, 9341730_00m_LEFT, 9342351_00m_LEFT, 9344414_00m_LEFT, 9344414_00m_RIGHT, 9346354_00m_RIGHT,

9351245_00m_RIGHT, 9351479_00m_LEFT, 9351479_00m_RIGHT, 9352437_00m_LEFT, 9352883_00m_LEFT, 9352883_12m_LEFT,

9352883_24m_LEFT, 9352883_36m_LEFT, 9353017_00m_RIGHT, 9353703_00m_RIGHT, 9354699_00m_RIGHT, 9354699_12m_RIGHT,

9354699_24m_RIGHT, 9354699_36m_RIGHT, 9354699_48m_RIGHT, 9355257_00m_RIGHT, 9356006_00m_RIGHT, 9356305_00m_RIGHT,

9357226_00m_RIGHT, 9357383_00m_LEFT, 9357856_00m_LEFT, 9357990_00m_RIGHT, 9358131_00m_RIGHT, 9358383_00m_RIGHT,

9360034_00m_RIGHT, 9360202_00m_LEFT, 9361302_00m_RIGHT, 9362264_00m_RIGHT, 9362660_00m_RIGHT, 9363397_00m_LEFT,

9364232_00m_RIGHT, 9364366_00m_RIGHT, 9365356_00m_LEFT, 9365356_12m_LEFT, 9365356_24m_LEFT, 9365968_00m_LEFT,

9367389_00m_RIGHT, 9367389_12m_RIGHT, 9367389_24m_RIGHT, 9367477_00m_RIGHT, 9367965_00m_RIGHT, 9368395_00m_RIGHT,

9368408_00m_RIGHT, 9369375_00m_LEFT, 9369649_00m_LEFT, 9371094_00m_RIGHT, 9371267_00m_RIGHT, 9372219_00m_RIGHT,

9373191_00m_LEFT, 9373786_00m_RIGHT, 9374235_00m_RIGHT, 9376280_00m_RIGHT, 9377726_00m_RIGHT, 9378009_00m_LEFT,

9379276_00m_RIGHT, 9379276_12m_RIGHT, 9381181_00m_LEFT, 9381777_00m_RIGHT, 9384183_00m_RIGHT, 9386991_00m_RIGHT,

9386991_12m_RIGHT, 9386991_24m_RIGHT, 9386991_36m_RIGHT, 9386991_48m_RIGHT, 9388068_00m_LEFT, 9390064_00m_LEFT,

9390205_00m_RIGHT, 9390312_00m_RIGHT, 9390719_00m_RIGHT, 9390995_00m_LEFT, 9391984_00m_RIGHT, 9392241_00m_LEFT,

9393127_00m_LEFT, 9393208_00m_RIGHT, 9393987_00m_RIGHT, 9394203_00m_RIGHT, 9396968_00m_RIGHT, 9396994_00m_LEFT,

9396994_00m_RIGHT, 9398272_00m_RIGHT, 9401202_00m_RIGHT, 9401202_12m_RIGHT, 9402055_00m_LEFT, 9402139_00m_LEFT,

9402139_00m_RIGHT, 9402139_12m_LEFT, 9402139_24m_LEFT, 9402960_00m_LEFT, 9403232_00m_LEFT, 9407018_00m_RIGHT,

9408506_00m_LEFT, 9409941_00m_RIGHT, 9409969_00m_LEFT, 9410941_00m_RIGHT, 9410941_12m_RIGHT, 9410941_24m_RIGHT,

9413039_00m_RIGHT, 9413071_00m_RIGHT, 9413071_12m_RIGHT, 9413071_24m_RIGHT, 9413071_36m_RIGHT, 9414083_00m_LEFT,

9414968_00m_LEFT, 9415069_00m_RIGHT, 9415074_00m_LEFT, 9415301_00m_RIGHT, 9416102_00m_LEFT, 9416308_00m_RIGHT,

9416381_00m_LEFT, 9416973_00m_LEFT, 9417063_00m_RIGHT, 9417307_00m_LEFT, 9417307_00m_RIGHT, 9417965_00m_RIGHT,

9418547_00m_RIGHT, 9420085_00m_LEFT, 9421389_00m_LEFT, 9421389_00m_RIGHT, 9421389_12m_LEFT, 9421389_24m_LEFT,

9421492_00m_LEFT, 9422075_00m_LEFT, 9422381_00m_RIGHT, 9423086_00m_RIGHT, 9428490_00m_RIGHT, 9429115_00m_RIGHT,

9429147_00m_RIGHT, 9429319_00m_RIGHT, 9429578_00m_RIGHT, 9430102_00m_RIGHT, 9432349_00m_LEFT, 9432349_00m_RIGHT,

9432349_12m_LEFT, 9432349_12m_RIGHT, 9432349_24m_LEFT, 9432349_24m_RIGHT, 9432349_36m_LEFT, 9432349_48m_LEFT,

9434150_00m_LEFT, 9435233_00m_RIGHT, 9435421_00m_RIGHT, 9436426_00m_LEFT, 9437111_00m_LEFT, 9438237_00m_LEFT,

9438523_00m_RIGHT, 9438852_00m_RIGHT, 9439411_00m_RIGHT, 9439428_00m_RIGHT, 9440307_00m_LEFT, 9441420_00m_LEFT,

9444411_00m_LEFT, 9445104_00m_RIGHT, 9445105_00m_RIGHT, 9448098_00m_RIGHT, 9448098_12m_RIGHT, 9448098_24m_RIGHT,

9448098_36m_RIGHT, 9448098_48m_RIGHT, 9448133_00m_LEFT, 9448133_00m_RIGHT, 9448315_00m_LEFT, 9451114_00m_LEFT,

9451499_00m_RIGHT, 9452100_00m_RIGHT, 9452239_00m_RIGHT, 9452305_00m_LEFT, 9452305_12m_LEFT, 9452305_24m_LEFT,

9453364_00m_RIGHT, 9453364_12m_RIGHT, 9453364_24m_RIGHT, 9454239_00m_LEFT, 9454239_00m_RIGHT, 9454239_12m_LEFT,

9454239_24m_LEFT, 9454360_00m_LEFT, 9457090_00m_RIGHT, 9457264_00m_RIGHT, 9457359_00m_LEFT, 9457359_00m_RIGHT,

9457359_12m_LEFT, 9457359_12m_RIGHT, 9457359_24m_LEFT, 9458107_00m_LEFT, 9458176_00m_LEFT, 9458176_12m_LEFT,

9458176_24m_LEFT, 9458176_36m_LEFT, 9458213_00m_RIGHT, 9458213_12m_RIGHT, 9458213_24m_RIGHT, 9458868_00m_LEFT,

9459295_00m_RIGHT, 9460225_00m_LEFT, 9460377_00m_RIGHT, 9461259_00m_RIGHT, 9461259_24m_RIGHT, 9461259_48m_RIGHT,

9463310_00m_LEFT, 9463326_00m_RIGHT, 9463326_12m_RIGHT, 9463326_24m_RIGHT, 9463326_36m_RIGHT, 9464409_00m_RIGHT,

9465287_00m_RIGHT, 9465298_00m_RIGHT, 9465413_00m_LEFT, 9465413_12m_LEFT, 9465413_24m_LEFT, 9465779_00m_RIGHT,

9467252_00m_LEFT, 9467252_12m_LEFT, 9467252_24m_LEFT, 9468250_00m_RIGHT, 9468292_00m_RIGHT, 9468561_00m_LEFT,

9469136_00m_RIGHT, 9470120_00m_LEFT, 9471143_00m_LEFT, 9471214_00m_LEFT, 9472327_00m_RIGHT, 9472327_12m_RIGHT,

9472327_24m_RIGHT, 9473302_00m_RIGHT, 9474248_00m_RIGHT, 9474571_00m_RIGHT, 9476283_00m_RIGHT, 9477205_00m_RIGHT,

9477293_00m_LEFT, 9477358_00m_RIGHT, 9478060_00m_LEFT, 9478504_12m_LEFT, 9478504_12m_RIGHT, 9478504_24m_LEFT,

9478504_24m_RIGHT, 9478504_36m_LEFT, 9478504_36m_RIGHT, 9478732_00m_LEFT, 9480215_00m_RIGHT, 9480215_12m_RIGHT,

9480480_00m_RIGHT, 9480636_00m_RIGHT, 9483254_00m_LEFT, 9483335_00m_LEFT, 9484333_00m_LEFT, 9485359_00m_LEFT,

9485359_12m_LEFT, 9485359_24m_LEFT, 9486466_00m_LEFT, 9486827_00m_LEFT, 9486952_00m_LEFT, 9487842_00m_RIGHT,

9488319_00m_LEFT, 9488360_00m_LEFT, 9488441_00m_RIGHT, 9489123_00m_LEFT, 9491365_00m_LEFT, 9491365_12m_LEFT,

9491365_24m_LEFT, 9491862_00m_RIGHT, 9493073_00m_LEFT, 9493856_00m_LEFT, 9493856_12m_LEFT, 9493856_24m_LEFT,

9494867_00m_RIGHT, 9495378_00m_LEFT, 9495873_00m_RIGHT, 9495873_12m_RIGHT, 9495873_24m_RIGHT, 9495873_36m_RIGHT,

9496436_00m_RIGHT, 9497534_00m_RIGHT, 9497534_12m_RIGHT, 9497534_24m_RIGHT, 9497734_00m_LEFT, 9498865_00m_LEFT,

9499341_00m_RIGHT, 9499893_00m_RIGHT, 9500225_00m_RIGHT, 9500365_00m_LEFT, 9500617_00m_RIGHT, 9502938_00m_LEFT,

9503347_00m_LEFT, 9504627_00m_RIGHT, 9504935_00m_RIGHT, 9505935_00m_LEFT, 9505935_12m_LEFT, 9505935_24m_LEFT,

9506155_00m_RIGHT, 9507886_00m_RIGHT, 9508335_00m_LEFT, 9509417_00m_RIGHT, 9509417_12m_RIGHT, 9510418_00m_RIGHT,

9510943_00m_LEFT, 9511423_00m_LEFT, 9511423_00m_RIGHT, 9511423_12m_LEFT, 9511423_12m_RIGHT, 9511423_24m_LEFT,

9511423_24m_RIGHT, 9511862_00m_RIGHT, 9513566_00m_LEFT, 9513860_00m_RIGHT, 9513860_12m_RIGHT, 9513860_24m_RIGHT,

9514956_00m_LEFT, 9516929_00m_LEFT, 9516929_12m_LEFT, 9516929_24m_LEFT, 9517311_00m_RIGHT, 9518267_00m_LEFT,

9518267_00m_RIGHT, 9519310_00m_LEFT, 9524432_00m_RIGHT, 9525952_00m_LEFT, 9525952_12m_LEFT, 9526396_00m_RIGHT,

9526396_12m_RIGHT, 9526396_24m_RIGHT, 9526739_00m_RIGHT, 9527954_00m_RIGHT, 9529676_00m_LEFT, 9530445_00m_LEFT,

9530445_12m_LEFT, 9530445_24m_LEFT, 9530445_36m_LEFT, 9530683_00m_LEFT, 9531454_00m_LEFT, 9531454_00m_RIGHT,

9531528_00m_RIGHT, 9532902_00m_LEFT, 9532970_00m_LEFT, 9532970_00m_RIGHT, 9532970_12m_LEFT, 9532970_12m_RIGHT,

9532970_24m_LEFT, 9532970_24m_RIGHT, 9532970_36m_LEFT, 9532970_48m_LEFT, 9534106_00m_RIGHT, 9534616_00m_RIGHT,

9534875_00m_LEFT, 9537090_00m_LEFT, 9537947_00m_LEFT, 9538599_00m_RIGHT, 9539084_00m_RIGHT, 9539368_00m_LEFT,

9539593_00m_RIGHT, 9539593_12m_RIGHT, 9539593_24m_RIGHT, 9539953_00m_LEFT, 9540125_00m_LEFT, 9540883_00m_RIGHT,

9540883_12m_LEFT, 9540883_12m_RIGHT, 9540883_24m_LEFT, 9540883_24m_RIGHT, 9541614_00m_RIGHT, 9542088_00m_LEFT,

9542889_00m_RIGHT, 9543202_00m_RIGHT, 9546643_00m_LEFT, 9547043_00m_RIGHT, 9547902_00m_LEFT, 9547902_12m_LEFT,

9547902_24m_LEFT, 9547902_36m_LEFT, 9548401_00m_RIGHT, 9549330_00m_LEFT, 9549405_00m_LEFT, 9549818_00m_RIGHT,

9549818_12m_RIGHT, 9549818_24m_RIGHT, 9551668_00m_LEFT, 9551857_00m_LEFT, 9551857_00m_RIGHT, 9551857_12m_LEFT,

9551857_12m_RIGHT, 9551857_24m_LEFT, 9551857_24m_RIGHT, 9552108_00m_LEFT, 9552108_00m_RIGHT, 9553149_00m_RIGHT,

9554631_00m_LEFT, 9554631_12m_LEFT, 9556402_00m_LEFT, 9556608_00m_RIGHT, 9557454_00m_RIGHT, 9560374_00m_LEFT,

9560374_00m_RIGHT, 9560374_12m_LEFT, 9560374_12m_RIGHT, 9560478_00m_RIGHT, 9560965_00m_LEFT, 9560965_00m_RIGHT,

9560965_12m_LEFT, 9560965_12m_RIGHT, 9560965_24m_LEFT, 9560965_24m_RIGHT, 9560965_36m_RIGHT, 9560965_48m_RIGHT,

9561824_00m_LEFT, 9561824_12m_LEFT, 9561824_24m_LEFT, 9562591_00m_LEFT, 9563176_00m_RIGHT, 9564189_00m_RIGHT,

9564509_00m_RIGHT, 9564509_12m_RIGHT, 9564509_24m_RIGHT, 9564729_00m_RIGHT, 9565192_00m_LEFT, 9566148_00m_RIGHT,

9566233_00m_RIGHT, 9566781_00m_RIGHT, 9566972_00m_RIGHT, 9567504_00m_RIGHT, 9567621_00m_LEFT, 9568706_00m_RIGHT,

9568938_00m_LEFT, 9568974_00m_LEFT, 9568974_00m_RIGHT, 9568974_12m_LEFT, 9568974_12m_RIGHT, 9568974_24m_LEFT,

9568974_24m_RIGHT, 9568974_36m_LEFT, 9569020_00m_RIGHT, 9571631_00m_LEFT, 9572192_00m_RIGHT, 9572632_00m_LEFT,

9572718_00m_RIGHT, 9572948_00m_RIGHT, 9574138_00m_RIGHT, 9575145_00m_RIGHT, 9575273_00m_RIGHT, 9576138_00m_RIGHT,

9577982_00m_RIGHT, 9578416_12m_LEFT, 9578416_24m_LEFT, 9578416_36m_LEFT, 9579255_00m_LEFT, 9580474_00m_RIGHT,

9581241_00m_RIGHT, 9581241_12m_RIGHT, 9581241_24m_RIGHT, 9581241_36m_RIGHT, 9581241_48m_RIGHT, 9581253_00m_LEFT,

9581253_00m_RIGHT, 9581712_00m_RIGHT, 9582223_00m_RIGHT, 9582223_12m_RIGHT, 9582223_24m_RIGHT, 9582223_36m_RIGHT,

9584990_00m_LEFT, 9584990_12m_LEFT, 9584990_24m_LEFT, 9585175_00m_LEFT, 9585218_00m_RIGHT, 9586270_00m_RIGHT,

9588436_00m_RIGHT, 9589708_00m_RIGHT, 9590145_00m_LEFT, 9590145_12m_LEFT, 9590145_24m_LEFT, 9590485_00m_RIGHT,

9590485_12m_RIGHT, 9590485_24m_RIGHT, 9590485_36m_RIGHT, 9592258_00m_RIGHT, 9592750_00m_LEFT, 9592750_12m_LEFT,

9592750_24m_LEFT, 9594608_00m_LEFT, 9594608_00m_RIGHT, 9595419_00m_LEFT, 9596238_00m_LEFT, 9596238_00m_RIGHT,

9596238_12m_LEFT, 9596238_12m_RIGHT, 9596238_24m_LEFT, 9596238_24m_RIGHT, 9596610_00m_LEFT, 9596610_12m_LEFT,

9597241_00m_LEFT, 9597241_00m_RIGHT, 9597680_00m_LEFT, 9597680_00m_RIGHT, 9598170_00m_LEFT, 9598170_12m_LEFT,

9598170_24m_LEFT, 9598683_00m_LEFT, 9599509_00m_RIGHT, 9599509_12m_RIGHT, 9599673_00m_RIGHT, 9601011_00m_LEFT,

9601011_12m_LEFT, 9601011_24m_LEFT, 9602668_00m_LEFT, 9602703_00m_RIGHT, 9603227_00m_RIGHT, 9604541_00m_RIGHT,

9604541_12m_RIGHT, 9604541_24m_RIGHT, 9604541_36m_RIGHT, 9606490_00m_LEFT, 9606490_00m_RIGHT, 9606664_00m_RIGHT,

9607739_00m_RIGHT, 9608253_00m_LEFT, 9608253_12m_LEFT, 9608253_24m_LEFT, 9608765_00m_RIGHT, 9611631_00m_LEFT,

9613135_00m_LEFT, 9613488_00m_LEFT, 9613993_00m_LEFT, 9613993_00m_RIGHT, 9613993_12m_LEFT, 9613993_24m_LEFT,

9613993_36m_LEFT, 9614934_00m_RIGHT, 9615420_00m_RIGHT, 9615507_00m_LEFT, 9615754_00m_LEFT, 9615754_12m_LEFT,

9615754_24m_LEFT, 9615754_36m_LEFT, 9615754_48m_LEFT, 9616501_00m_RIGHT, 9617213_00m_RIGHT, 9618705_00m_LEFT,

9619405_00m_RIGHT, 9619698_00m_LEFT, 9619698_24m_LEFT, 9619698_48m_LEFT, 9619739_00m_LEFT, 9621208_00m_LEFT,

9621601_00m_LEFT, 9621601_12m_LEFT, 9621601_24m_LEFT, 9626098_00m_LEFT, 9626098_00m_RIGHT, 9626197_00m_LEFT,

9626197_12m_LEFT, 9626197_24m_LEFT, 9626197_48m_LEFT, 9627172_00m_RIGHT, 9627172_12m_RIGHT, 9627172_24m_RIGHT,

9627197_00m_RIGHT, 9627716_00m_RIGHT, 9628194_00m_RIGHT, 9628194_12m_RIGHT, 9628194_24m_RIGHT, 9628194_36m_RIGHT,

9628227_00m_LEFT, 9628227_12m_LEFT, 9628436_00m_RIGHT, 9628649_00m_RIGHT, 9629158_00m_LEFT, 9629890_00m_RIGHT,

9631713_00m_RIGHT, 9631713_12m_RIGHT, 9631713_24m_RIGHT, 9631930_00m_LEFT, 9631930_12m_LEFT, 9631930_24m_LEFT,

9631930_36m_LEFT, 9631930_48m_LEFT, 9634187_00m_RIGHT, 9634187_12m_RIGHT, 9634187_24m_RIGHT, 9634422_00m_LEFT,

9634422_00m_RIGHT, 9634422_12m_LEFT, 9634422_12m_RIGHT, 9634422_24m_LEFT, 9634422_24m_RIGHT, 9635177_00m_RIGHT,

9635581_00m_RIGHT, 9635581_12m_RIGHT, 9635632_00m_RIGHT, 9636064_00m_RIGHT, 9637053_00m_LEFT, 9637958_00m_RIGHT,

9638953_00m_RIGHT, 9638953_12m_RIGHT, 9638953_24m_RIGHT, 9638953_36m_RIGHT, 9639088_00m_LEFT, 9639088_00m_RIGHT,

9639388_00m_LEFT, 9640473_00m_LEFT, 9640473_12m_LEFT, 9640473_24m_LEFT, 9641648_00m_LEFT, 9641648_00m_RIGHT,

9642695_00m_LEFT, 9642695_12m_LEFT, 9642695_24m_LEFT, 9642695_36m_LEFT, 9643944_00m_RIGHT, 9643944_12m_RIGHT,

9644109_00m_RIGHT, 9645478_00m_RIGHT, 9645478_12m_RIGHT, 9645478_24m_RIGHT, 9645683_00m_LEFT, 9646127_00m_RIGHT,

9646958_00m_LEFT, 9646958_00m_RIGHT, 9646958_12m_LEFT, 9646958_24m_LEFT, 9650272_00m_RIGHT, 9650705_00m_LEFT,

9651214_00m_RIGHT, 9653075_00m_RIGHT, 9653221_00m_RIGHT, 9653465_00m_LEFT, 9653659_00m_LEFT, 9654702_00m_RIGHT,

9657464_00m_LEFT, 9657594_00m_LEFT, 9657594_12m_LEFT, 9657594_24m_LEFT, 9658955_00m_RIGHT, 9659472_00m_LEFT,

9660708_00m_RIGHT, 9660708_12m_RIGHT, 9660708_24m_RIGHT, 9660708_36m_RIGHT, 9661460_00m_RIGHT, 9662427_00m_LEFT,

9662965_00m_LEFT, 9662965_12m_LEFT, 9662965_24m_LEFT, 9663463_00m_LEFT, 9663463_12m_LEFT, 9663463_24m_LEFT,

9663706_00m_RIGHT, 9664468_00m_LEFT, 9665234_00m_RIGHT, 9667081_00m_LEFT, 9667081_00m_RIGHT, 9668241_00m_LEFT,

9669735_00m_RIGHT, 9670965_00m_LEFT, 9670965_12m_LEFT, 9670965_24m_LEFT, 9672436_00m_RIGHT, 9672573_00m_LEFT,

9672573_00m_RIGHT, 9676101_00m_LEFT, 9676101_12m_LEFT, 9676101_24m_LEFT, 9676587_00m_RIGHT, 9679595_00m_RIGHT,

9679595_24m_RIGHT, 9680194_00m_RIGHT, 9680344_00m_LEFT, 9680591_00m_RIGHT, 9681696_00m_LEFT, 9681696_24m_LEFT,

9681696_48m_LEFT, 9681803_00m_RIGHT, 9681803_12m_RIGHT, 9681803_24m_RIGHT, 9682600_00m_LEFT, 9683704_00m_RIGHT,

9683736_00m_RIGHT, 9684122_00m_LEFT, 9684822_00m_LEFT, 9685077_00m_LEFT, 9686327_00m_RIGHT, 9686777_00m_LEFT,

9686777_24m_LEFT, 9687779_00m_LEFT, 9687779_00m_RIGHT, 9687779_12m_LEFT, 9687779_24m_LEFT, 9688649_00m_LEFT,

9689922_00m_LEFT, 9689922_00m_RIGHT, 9689922_12m_RIGHT, 9690400_00m_RIGHT, 9690658_00m_RIGHT, 9690658_12m_RIGHT,

9690658_24m_RIGHT, 9690658_36m_RIGHT, 9690658_48m_RIGHT, 9691663_00m_LEFT, 9691663_12m_LEFT, 9691663_24m_LEFT,

9691663_36m_LEFT, 9691663_48m_LEFT, 9692417_00m_RIGHT, 9692417_12m_RIGHT, 9692417_24m_RIGHT, 9693161_00m_LEFT,

9693161_12m_LEFT, 9693161_24m_LEFT, 9693364_00m_LEFT, 9693806_00m_LEFT, 9693806_00m_RIGHT, 9695686_00m_LEFT,

9695962_00m_RIGHT, 9696350_00m_RIGHT, 9699448_00m_LEFT, 9699448_00m_RIGHT, 9704858_00m_RIGHT, 9706468_00m_LEFT,

9706468_00m_RIGHT, 9706468_12m_LEFT, 9706468_12m_RIGHT, 9706468_24m_LEFT, 9706468_24m_RIGHT, 9707649_00m_RIGHT,

9708289_00m_LEFT, 9708289_00m_RIGHT, 9708289_12m_RIGHT, 9708289_24m_RIGHT, 9708289_36m_RIGHT, 9708289_48m_RIGHT,

9708353_00m_RIGHT, 9708759_00m_LEFT, 9708759_00m_RIGHT, 9708759_12m_LEFT, 9708759_12m_RIGHT, 9708759_24m_LEFT,

9708759_24m_RIGHT, 9708759_36m_LEFT, 9711284_00m_RIGHT, 9711687_00m_LEFT, 9711687_00m_RIGHT, 9712471_00m_LEFT,

9712471_00m_RIGHT, 9712471_24m_LEFT, 9712471_24m_RIGHT, 9712471_36m_LEFT, 9712471_48m_LEFT, 9712762_00m_LEFT, 9716984_00m_RIGHT, 9716984_12m_RIGHT, 9716984_24m_RIGHT, 9718322_00m_LEFT, 9719999_00m_RIGHT, 9721540_00m_LEFT, 9721540_12m_LEFT, 9723575_00m_RIGHT, 9724286_00m_LEFT, 9724286_00m_RIGHT, 9724286_12m_LEFT, 9724286_12m_RIGHT, 9724286_24m_LEFT, 9724286_24m_RIGHT, 9724286_36m_RIGHT, 9724907_00m_RIGHT, 9726778_00m_LEFT, 9726778_12m_LEFT, 9727780_00m_RIGHT, 9727984_00m_LEFT, 9729306_00m_RIGHT, 9731208_00m_RIGHT, 9732727_00m_RIGHT, 9732727_12m_RIGHT, 9732727_24m_RIGHT, 9732751_00m_RIGHT, 9733288_00m_RIGHT, 9735403_00m_RIGHT, 9736290_00m_LEFT, 9736290_00m_RIGHT, 9736290_12m_LEFT, 9736290_12m_RIGHT, 9736290_24m_LEFT, 9736290_24m_RIGHT, 9739777_00m_RIGHT, 9741448_00m_RIGHT, 9742871_00m_LEFT, 9742871_00m_RIGHT, 9742871_12m_LEFT, 9742871_12m_RIGHT, 9743052_00m_LEFT, 9744444_00m_RIGHT, 9745458_00m_RIGHT, 9745458_12m_RIGHT, 9745458_24m_RIGHT, 9745458_36m_RIGHT, 9745596_00m_LEFT, 9745596_12m_LEFT, 9745596_24m_LEFT, 9746075_00m_RIGHT, 9746090_00m_RIGHT, 9746090_12m_RIGHT, 9746090_24m_RIGHT, 9746467_00m_RIGHT, 9750582_00m_RIGHT, 9751401_00m_RIGHT, 9751625_00m_RIGHT, 9755634_00m_RIGHT, 9755935_00m_RIGHT, 9755935_12m_RIGHT, 9759942_00m_RIGHT, 9759956_00m_RIGHT, 9760079_00m_RIGHT, 9760954_00m_RIGHT, 9761431_00m_RIGHT, 9761463_00m_RIGHT, 9762600_00m_RIGHT, 9762973_00m_LEFT, 9763679_00m_RIGHT, 9764936_00m_RIGHT, 9766202_00m_RIGHT, 9766939_00m_LEFT, 9768500_00m_LEFT, 9770217_00m_LEFT, 9771727_00m_LEFT, 9772692_00m_LEFT, 9774237_00m_RIGHT, 9775951_00m_LEFT, 9777033_00m_LEFT, 9777777_00m_LEFT, 9778518_00m_LEFT, 9780275_00m_RIGHT, 9785243_00m_RIGHT, 9785243_24m_RIGHT, 9785331_00m_RIGHT, 9785331_12m_RIGHT, 9785331_24m_RIGHT, 9785978_00m_LEFT, 9786086_00m_LEFT, 9786086_12m_LEFT, 9786086_24m_LEFT, 9786946_00m_RIGHT, 9787693_00m_RIGHT, 9787693_12m_RIGHT, 9787693_24m_RIGHT, 9788072_00m_LEFT, 9789095_00m_RIGHT, 9790623_00m_LEFT, 9791051_00m_LEFT, 9791051_36m_LEFT, 9792220_00m_RIGHT, 9793168_00m_RIGHT, 9793845_00m_LEFT, 9796889_00m_RIGHT, 9797127_00m_RIGHT, 9797352_00m_LEFT, 9797352_00m_RIGHT, 9797352_12m_LEFT, 9797352_12m_RIGHT, 9797352_24m_LEFT, 9797352_24m_RIGHT, 9797352_36m_RIGHT, 9797791_00m_RIGHT, 9799305_00m_LEFT, 9799900_00m_LEFT, 9799900_12m_LEFT, 9802320_00m_RIGHT, 9802877_00m_LEFT, 9803694_00m_LEFT, 9804361_00m_LEFT, 9804361_12m_LEFT, 9804361_24m_LEFT, 9804361_36m_LEFT, 9804361_48m_LEFT, 9805321_00m_RIGHT, 9809967_00m_LEFT, 9809967_00m_RIGHT, 9811200_00m_RIGHT, 9811475_00m_LEFT, 9811840_00m_RIGHT, 9812168_00m_LEFT, 9812168_12m_LEFT, 9812168_24m_LEFT, 9813780_00m_RIGHT, 9815779_00m_LEFT, 9815779_12m_LEFT, 9815779_24m_LEFT, 9816736_00m_RIGHT, 9817241_00m_RIGHT, 9817661_00m_RIGHT, 9818359_00m_LEFT, 9819273_00m_LEFT, 9820479_00m_LEFT, 9821265_00m_LEFT, 9821982_00m_RIGHT, 9824005_00m_LEFT, 9824005_12m_LEFT, 9824274_00m_LEFT, 9832481_00m_RIGHT, 9832566_00m_LEFT, 9832566_00m_RIGHT, 9832566_12m_LEFT, 9832566_12m_RIGHT, 9832566_24m_LEFT, 9832566_24m_RIGHT, 9832566_36m_LEFT, 9832566_36m_RIGHT, 9832566_48m_RIGHT, 9833489_00m_LEFT, 9833782_00m_LEFT, 9834325_00m_LEFT, 9835852_00m_RIGHT, 9838045_00m_RIGHT, 9838045_12m_RIGHT, 9838045_24m_RIGHT, 9840837_00m_RIGHT, 9841033_00m_LEFT, 9841033_00m_RIGHT, 9843822_00m_RIGHT, 9844838_00m_RIGHT, 9845361_00m_LEFT, 9846239_00m_LEFT, 9847354_00m_LEFT, 9847541_00m_RIGHT, 9847541_12m_RIGHT, 9847541_24m_RIGHT, 9847541_36m_RIGHT, 9847873_00m_LEFT, 9847873_00m_RIGHT, 9847873_12m_LEFT,

9847873_24m_LEFT, 9848239_00m_RIGHT, 9850238_00m_RIGHT, 9850238_12m_RIGHT, 9850238_24m_RIGHT, 9850238_36m_RIGHT, 9850865_00m_LEFT, 9850865_00m_RIGHT, 9851758_00m_RIGHT, 9852668_00m_LEFT, 9858216_00m_RIGHT, 9858840_00m_RIGHT, 9860552_00m_RIGHT, 9864733_00m_LEFT, 9866244_00m_LEFT, 9866291_00m_LEFT, 9866291_12m_LEFT, 9866291_24m_LEFT, 9867284_00m_LEFT, 9867284_12m_LEFT, 9867284_24m_LEFT, 9867315_00m_LEFT, 9870569_00m_LEFT, 9870569_00m_RIGHT, 9871046_00m_LEFT, 9871046_12m_LEFT, 9871046_24m_LEFT, 9872241_00m_RIGHT, 9872810_00m_RIGHT, 9872810_12m_RIGHT, 9872810_24m_RIGHT, 9874784_00m_RIGHT, 9874820_00m_LEFT, 9875303_00m_LEFT, 9877597_00m_LEFT, 9878594_00m_LEFT, 9878594_00m_RIGHT, 9878594_12m_LEFT, 9878594_24m_LEFT, 9879774_00m_RIGHT, 9884591_00m_RIGHT, 9886575_00m_RIGHT, 9887519_00m_RIGHT, 9887519_12m_RIGHT, 9887519_24m_RIGHT, 9887519_36m_RIGHT, 9887519_48m_RIGHT, 9887703_00m_RIGHT, 9888515_00m_RIGHT, 9889762_00m_RIGHT, 9889762_12m_RIGHT, 9889762_36m_RIGHT, 9895555_00m_LEFT, 9899098_00m_RIGHT, 9899098_12m_RIGHT, 9899098_24m_RIGHT, 9903160_00m_RIGHT, 9903394_00m_LEFT, 9904574_00m_LEFT, 9905156_00m_LEFT, 9905156_00m_RIGHT, 9905156_12m_RIGHT, 9905156_24m_RIGHT, 9905276_00m_RIGHT, 9906008_00m_LEFT, 9906008_00m_RIGHT, 9907800_00m_LEFT, 9907909_00m_LEFT, 9908796_00m_RIGHT, 9910391_00m_LEFT, 9910391_00m_RIGHT, 9910391_12m_RIGHT, 9910391_24m_RIGHT, 9910719_00m_LEFT, 9911221_00m_RIGHT, 9916496_00m_RIGHT, 9916542_00m_RIGHT, 9917284_00m_LEFT, 9917284_00m_RIGHT, 9917505_00m_RIGHT, 9917803_00m_LEFT, 9917803_12m_LEFT, 9919568_00m_LEFT, 9920140_00m_RIGHT, 9920298_00m_LEFT, 9920298_00m_RIGHT, 9920684_00m_RIGHT, 9920684_24m_RIGHT, 9920684_48m_RIGHT, 9921811_00m_RIGHT, 9922855_00m_LEFT, 9922855_00m_RIGHT, 9922855_12m_LEFT, 9922855_12m_RIGHT, 9922855_24m_LEFT, 9922855_24m_RIGHT, 9922855_36m_LEFT, 9922855_36m_RIGHT, 9922855_48m_LEFT, 9922855_48m_RIGHT, 9925594_00m_LEFT, 9925594_00m_RIGHT, 9926602_00m_RIGHT, 9926839_00m_LEFT, 9927318_00m_LEFT, 9928082_00m_LEFT, 9928272_00m_RIGHT, 9928272_12m_RIGHT, 9928272_24m_RIGHT, 9928319_00m_LEFT, 9928319_12m_LEFT, 9928319_24m_LEFT, 9930342_00m_RIGHT, 9930342_12m_RIGHT, 9932333_00m_RIGHT, 9932333_12m_RIGHT, 9932809_00m_LEFT, 9933459_00m_LEFT, 9936451_00m_RIGHT, 9937239_00m_RIGHT, 9938236_00m_RIGHT, 9939547_00m_LEFT, 9939718_00m_LEFT, 9940023_00m_RIGHT, 9942319_00m_LEFT, 9942319_12m_LEFT, 9942319_24m_LEFT, 9943227_00m_LEFT, 9943227_00m_RIGHT, 9943818_00m_LEFT, 9946330_00m_RIGHT, 9946846_00m_RIGHT, 9946846_12m_RIGHT, 9947240_00m_RIGHT, 9949321_00m_RIGHT, 9951449_00m_RIGHT, 9952664_00m_RIGHT, 9954040_00m_LEFT, 9955365_00m_RIGHT, 9958220_00m_RIGHT, 9958220_12m_RIGHT, 9958220_24m_RIGHT, 9958234_00m_RIGHT, 9960314_00m_LEFT, 9960314_00m_RIGHT, 9961951_00m_LEFT, 9962029_00m_RIGHT, 9962749_00m_RIGHT, 9962749_12m_RIGHT, 9962749_24m_RIGHT, 9964232_00m_RIGHT, 9965231_00m_RIGHT, 9965231_12m_RIGHT, 9966343_00m_RIGHT, 9967211_00m_RIGHT, 9967358_00m_RIGHT, 9967358_12m_RIGHT, 9967358_24m_RIGHT, 9967358_36m_RIGHT, 9967358_48m_RIGHT, 9967719_00m_LEFT, 9967719_12m_LEFT, 9967719_24m_LEFT, 9967728_00m_LEFT, 9967728_12m_LEFT, 9967728_24m_LEFT, 9968283_00m_LEFT, 9968721_00m_LEFT, 9968924_00m_LEFT, 9968924_12m_LEFT, 9968924_24m_LEFT, 9968924_36m_LEFT, 9968924_48m_LEFT, 9969009_00m_LEFT, 9973713_00m_LEFT, 9973713_12m_LEFT, 9973713_24m_LEFT, 9975485_00m_RIGHT, 9975485_12m_RIGHT, 9975485_24m_RIGHT, 9975485_36m_RIGHT, 9976207_00m_LEFT, 9976720_00m_LEFT, 9976720_12m_LEFT, 9976720_24m_LEFT, 9977985_00m_RIGHT,

9978026_00m_LEFT, 9978026_12m_LEFT, 9978026_24m_LEFT, 9981798_00m_RIGHT, 9982186_00m_LEFT, 9985277_00m_LEFT, 9985803_00m_LEFT, 9986355_00m_RIGHT, 9988027_00m_LEFT, 9988891_00m_RIGHT, 9988891_12m_RIGHT, 9989309_00m_LEFT, 9989700_00m_RIGHT, 9991313_00m_LEFT, 9991313_12m_LEFT, 9991313_24m_LEFT, 9991313_36m_LEFT, 9991580_00m_LEFT, 9992358_00m_RIGHT, 9992358_12m_RIGHT, 9992358_24m_RIGHT, 9993650_00m_LEFT, 9993650_12m_RIGHT, 9993833_00m_LEFT, 9994408_00m_RIGHT, 9995338_00m_LEFT, 9996098_00m_RIGHT, 9996865_00m_LEFT, 9996865_00m_RIGHT, 9997381_00m_RIGHT, 9997856_00m_LEFT.

## MOST Database

The ID, follow-up information, and knee side are provided below. V0: baseline, V1: 15 months, V2: 30 months, and V3: 60 months follow-up data.

## MOST Database Sagittal TSE MRI Data:

M0233_V0_RIGHT, M2183_V0_RIGHT, M0327_V0_LEFT, M2119_V0_LEFT, M2166_V0_LEFT, M0620_V0_LEFT, M1711_V0_LEFT, M1811_V0_LEFT, M0759_V0_LEFT, M2305_V0_RIGHT, M2259_V0_LEFT, M0638_V0_RIGHT, M2120_V0_RIGHT, M0734_V0_RIGHT, M1101_V0_RIGHT, M0372_V0_LEFT, M1005_V0_LEFT, M1980_V0_LEFT, M2334_V0_RIGHT, M0058_V0_LEFT, M0381_V0_RIGHT, M1897_V0_LEFT, M2170_V0_RIGHT, M2286_V0_RIGHT, M0015_V0_LEFT, M0968_V0_LEFT, M1241_V0_LEFT, M1749_V0_RIGHT, M2423_V0_LEFT, M0053_V0_LEFT, M1238_V0_RIGHT, M2045_V0_LEFT, M0661_V0_LEFT, M1014_V0_RIGHT, M0187_V0_LEFT, M1186_V0_LEFT, M1402_V0_RIGHT, M1526_V0_LEFT, M1955_V0_LEFT, M0229_V0_RIGHT, M0371_V0_RIGHT, M0399_V0_LEFT, M1502_V0_RIGHT, M1522_V0_LEFT, M2183_V1_RIGHT, M2243_V0_LEFT, M2687_V0_LEFT, M2920_V0_LEFT, M0905_V0_RIGHT, M0938_V0_LEFT, M1299_V0_RIGHT, M1913_V0_LEFT, M2788_V0_LEFT, M0576_V0_LEFT, M1894_V0_RIGHT, M2225_V0_LEFT, M2361_V0_LEFT, M2446_V0_RIGHT, M2561_V0_RIGHT, M1104_V0_LEFT, M1375_V0_RIGHT, M1995_V0_RIGHT, M2995_V0_RIGHT, M0066_V0_LEFT, M0123_V0_LEFT, M1811_V1_LEFT, M1912_V0_RIGHT, M0504_V0_LEFT, M0541_V0_RIGHT, M0759_V1_LEFT, M2560_V0_LEFT, M0090_V0_LEFT, M0136_V0_LEFT, M0700_V0_RIGHT, M2356_V0_RIGHT, M0601_V0_RIGHT, M0638_V1_RIGHT, M1305_V0_RIGHT, M1448_V0_RIGHT, M2031_V0_LEFT, M2271_V0_LEFT, M2737_V0_RIGHT, M0100_V0_RIGHT, M0231_V0_RIGHT, M0723_V0_RIGHT, M1570_V0_RIGHT, M1656_V0_RIGHT, M2568_V0_RIGHT, M0044_V0_LEFT, M0648_V0_LEFT, M0907_V0_LEFT, M2323_V0_LEFT, M2849_V2_LEFT, M0058_V1_LEFT, M0408_V0_LEFT, M0707_V0_RIGHT, M0994_V0_RIGHT, M1202_V0_RIGHT, M1377_V0_LEFT, M1516_V0_LEFT, M1617_V0_RIGHT, M1897_V1_LEFT, M2264_V0_RIGHT, M2286_V1_RIGHT, M1691_V0_RIGHT, M2094_V0_RIGHT, M2415_V0_RIGHT, M3018_V0_RIGHT, M0479_V0_LEFT, M1735_V0_RIGHT, M2476_V0_RIGHT, M0661_V1_LEFT, M1151_V0_RIGHT, M2110_V0_RIGHT, M2731_V0_LEFT, M0331_V0_RIGHT, M0511_V0_LEFT, M1402_V1_RIGHT, M1836_V0_LEFT, M2339_V0_LEFT, M0233_V3_RIGHT, M0371_V1_RIGHT, M0814_V0_RIGHT, M1631_V0_RIGHT, M1774_V0_LEFT, M1788_V0_RIGHT, M2183_V2_RIGHT, M0019_V0_RIGHT, M0327_V2_LEFT, M0799_V0_RIGHT, M0938_V1_LEFT, M1300_V0_RIGHT, M2124_V0_RIGHT,

M1894_V1_RIGHT, M2119_V2_LEFT, M2166_V2_LEFT, M2606_V0_LEFT, M0333_V0_LEFT, M0658_V0_RIGHT, M1995_V1_RIGHT,

M0123_V1_LEFT, M0964_V0_LEFT, M1423_V0_LEFT, M1711_V2_LEFT, M1811_V2_LEFT, M0759_V2_LEFT, M1648_V0_LEFT,

M2305_V2_RIGHT, M2560_V1_LEFT, M2775_V1_LEFT, M0605_V0_LEFT, M1097_V0_LEFT, M1314_V0_RIGHT, M1457_V0_LEFT,

M2259_V2_LEFT, M2353_V2_LEFT, M2958_V0_LEFT, M2978_V0_RIGHT, M0134_V0_RIGHT, M0291_V0_RIGHT, M0638_V2_RIGHT,

M1305_V1_RIGHT, M1467_V0_LEFT, M2081_V0_LEFT, M2120_V2_RIGHT, M0247_V0_RIGHT, M0723_V1_RIGHT, M0734_V2_RIGHT,

M0958_V0_RIGHT, M1101_V2_RIGHT, M1549_V0_LEFT, M1979_V0_RIGHT, M2103_V0_LEFT, M2357_V0_RIGHT, M2568_V1_RIGHT,

M2718_V0_RIGHT, M3001_V0_LEFT, M0372_V2_LEFT, M1005_V2_LEFT, M1980_V2_LEFT, M2839_V0_LEFT, M0058_V2_LEFT,

M0381_V2_RIGHT, M0774_V0_RIGHT, M1897_V2_LEFT, M2170_V2_RIGHT, M2817_V0_LEFT, M0015_V2_LEFT, M0101_V0_RIGHT,

M1241_V2_LEFT, M1486_V0_LEFT, M1749_V2_RIGHT, M1903_V0_RIGHT, M2423_V2_LEFT, M0053_V2_LEFT, M0434_V0_RIGHT,

M1238_V2_RIGHT, M1641_V0_RIGHT, M2045_V2_LEFT, M2556_V1_RIGHT, M0529_V0_LEFT, M0661_V2_LEFT, M0904_V0_LEFT,

M1014_V2_RIGHT, M1246_V0_LEFT, M1947_V0_RIGHT, M2800_V0_RIGHT, M2872_V0_RIGHT, M0187_V2_LEFT, M1045_V0_RIGHT,

M1186_V2_LEFT, M1402_V2_RIGHT, M1411_V0_LEFT, M1548_V0_LEFT, M1849_V0_RIGHT, M1955_V2_LEFT, M2339_V1_LEFT,

M0229_V2_RIGHT, M0371_V2_RIGHT, M0399_V2_LEFT, M0720_V0_LEFT, M1502_V2_RIGHT, M1522_V2_LEFT, M1774_V1_LEFT,

M2243_V2_LEFT, M2687_V2_LEFT, M2920_V2_LEFT, M0413_V0_LEFT, M0713_V0_RIGHT, M0799_V1_RIGHT, M0938_V2_LEFT,

M1299_V2_RIGHT, M1572_V0_RIGHT, M1913_V2_LEFT, M2067_V0_LEFT, M2430_V0_RIGHT, M0241_V0_RIGHT, M0936_V0_LEFT,

M1894_V2_RIGHT, M2361_V2_LEFT, M2446_V2_RIGHT, M2561_V2_RIGHT, M2741_V0_RIGHT, M0303_V0_RIGHT, M1104_V2_LEFT,

M1375_V2_RIGHT, M1837_V0_LEFT, M1995_V2_RIGHT, M2995_V2_RIGHT, M0066_V2_LEFT, M0123_V2_LEFT, M0693_V0_RIGHT,

M0861_V0_LEFT, M1912_V2_RIGHT, M2014_V0_LEFT, M2281_V0_LEFT, M0201_V0_RIGHT, M0324_V0_LEFT, M0504_V2_LEFT,

M0541_V2_RIGHT, M1021_V0_RIGHT, M1778_V0_RIGHT, M2541_V0_RIGHT, M2560_V2_LEFT, M2775_V2_LEFT, M0090_V2_LEFT,

M0136_V2_LEFT, M0175_V0_RIGHT, M0700_V2_RIGHT, M1507_V1_RIGHT, M1674_V0_RIGHT, M1869_V0_LEFT, M2266_V0_RIGHT,

M2356_V2_RIGHT, M0134_V1_RIGHT, M0601_V2_RIGHT, M1448_V2_RIGHT, M1527_V0_LEFT, M2031_V2_LEFT, M2150_V0_RIGHT,

M2271_V2_LEFT, M2273_V0_LEFT, M2569_V2_LEFT, M2722_V0_RIGHT, M2737_V2_RIGHT, M2781_V0_RIGHT, M0100_V2_RIGHT,

M0723_V2_RIGHT, M0767_V0_RIGHT, M0810_V0_RIGHT, M1570_V2_RIGHT, M1656_V2_RIGHT, M1717_V0_RIGHT, M2568_V2_RIGHT,

M2900_V0_RIGHT, M2957_V0_RIGHT, M2998_V0_RIGHT, M3001_V1_LEFT, M0044_V2_LEFT, M0578_V0_RIGHT, M0648_V2_LEFT,

M1064_V0_LEFT, M1128_V0_LEFT, M1391_V0_RIGHT, M2323_V2_LEFT, M2341_V0_LEFT, M0011_V0_RIGHT, M0408_V2_LEFT,

M0707_V2_RIGHT, M0994_V2_RIGHT, M1120_V0_LEFT, M1202_V2_RIGHT, M1377_V2_LEFT, M1516_V2_LEFT, M1617_V2_RIGHT,

M2264_V2_RIGHT, M2308_V2_RIGHT, M2490_V0_LEFT, M2728_V0_LEFT, M2855_V0_LEFT, M3021_V0_LEFT, M1339_V0_RIGHT,

M1512_V0_LEFT, M1691_V2_RIGHT, M2192_V0_LEFT, M2415_V2_RIGHT, M2754_V0_LEFT, M3018_V2_RIGHT, M0479_V2_LEFT,

M0512_V0_LEFT, M0550_V0_LEFT, M0961_V0_RIGHT, M1198_V0_LEFT, M1735_V2_RIGHT, M2476_V2_RIGHT, M2556_V2_RIGHT,

M0016_V0_LEFT, M0098_V0_RIGHT, M0163_V0_RIGHT, M0264_V0_LEFT, M0273_V0_LEFT, M0904_V1_LEFT, M1360_V0_LEFT,

M1362_V2_RIGHT, M1745_V0_LEFT, M1804_V0_RIGHT, M1991_V0_RIGHT, M2072_V0_RIGHT, M2110_V2_RIGHT, M2307_V0_LEFT,

M2731_V2_LEFT, M2870_V0_LEFT, M0319_V0_RIGHT, M0457_V0_RIGHT, M0511_V2_LEFT, M0571_V0_LEFT, M0899_V0_LEFT,

M1184_V0_RIGHT, M1533_V0_RIGHT, M1836_V2_LEFT, M1849_V1_RIGHT, M2229_V0_RIGHT, M2339_V2_LEFT, M2650_V0_LEFT,

M0205_V0_RIGHT, M0716_V0_RIGHT, M0814_V2_RIGHT, M1171_V0_LEFT, M1631_V2_RIGHT, M1774_V2_LEFT, M1788_V2_RIGHT,

M1854_V0_RIGHT, M1942_V0_LEFT, M2183_V3_RIGHT, M2750_V0_RIGHT, M2964_V0_RIGHT, M0019_V2_RIGHT, M0327_V3_LEFT,

M0799_V2_RIGHT, M1237_V0_RIGHT, M1300_V2_RIGHT, M1393_V2_RIGHT, M1707_V0_LEFT, M2067_V1_LEFT, M2124_V2_RIGHT,

M2256_V0_LEFT, M2607_V0_RIGHT, M2735_V0_LEFT, M0241_V1_RIGHT, M0808_V0_LEFT, M1390_V0_RIGHT, M1567_V0_RIGHT,

M2689_V0_LEFT, M0032_V0_RIGHT, M0033_V0_LEFT, M0658_V2_RIGHT, M0999_V0_RIGHT, M1834_V0_LEFT, M1948_V0_RIGHT,

M0203_V0_RIGHT, M0693_V1_RIGHT, M0964_V2_LEFT, M0967_V0_LEFT, M1423_V2_LEFT, M1711_V3_LEFT, M1811_V3_LEFT,

M2347_V0_RIGHT, M0667_V0_LEFT, M0716_V3_LEFT, M0815_V0_RIGHT, M1021_V1_RIGHT, M1233_V0_LEFT, M1238_V3_LEFT,

M1294_V0_LEFT, M1625_V0_LEFT, M1648_V2_LEFT, M2048_V0_LEFT, M2442_V0_LEFT, M2552_V0_LEFT, M2715_V1_RIGHT,

M2744_V0_RIGHT, M2768_V0_LEFT, M0268_V0_LEFT, M0605_V2_LEFT, M0903_V0_LEFT, M1314_V2_RIGHT, M1457_V2_LEFT,

M1507_V2_RIGHT, M2259_V3_LEFT, M2266_V1_RIGHT, M2353_V3_LEFT, M2958_V2_LEFT, M2978_V2_RIGHT, M3026_V0_LEFT,

M0134_V2_RIGHT, M0176_V0_RIGHT, M0638_V3_RIGHT, M1467_V2_LEFT, M1497_V0_RIGHT, M2081_V2_LEFT, M2120_V3_RIGHT,

M2270_V0_LEFT, M0247_V2_RIGHT, M0734_V3_RIGHT, M0844_V0_RIGHT, M0958_V2_RIGHT, M1089_V0_LEFT, M1101_V3_RIGHT,

M1549_V2_LEFT, M1979_V2_RIGHT, M2058_V0_RIGHT, M2103_V2_LEFT, M2357_V2_RIGHT, M2718_V2_RIGHT, M2848_V0_LEFT,

M2957_V1_RIGHT, M3001_V2_LEFT, M1005_V3_LEFT, M1340_V0_RIGHT, M1965_V0_RIGHT, M1980_V3_LEFT, M2022_V0_RIGHT,

M2778_V0_LEFT, M2839_V2_LEFT, M0058_V3_LEFT, M0774_V2_RIGHT, M1747_V0_LEFT, M2170_V3_RIGHT, M2817_V2_LEFT,

M0015_V3_LEFT, M0053_V3_RIGHT, M0827_V0_RIGHT, M1177_V2_RIGHT, M1243_V0_LEFT, M1486_V2_LEFT, M1643_V0_RIGHT,

M1684_V2_LEFT, M1747_V2_RIGHT, M1835_V0_LEFT, M1903_V2_RIGHT, M2423_V3_LEFT, M2849_V0_RIGHT, M0022_V0_RIGHT,

M0434_V2_RIGHT, M0470_V0_LEFT, M0550_V1_LEFT, M0588_V0_RIGHT, M1198_V1_LEFT, M1604_V0_RIGHT, M1641_V2_RIGHT,

M2045_V3_LEFT, M0098_V1_RIGHT, M0264_V1_LEFT, M0529_V2_LEFT, M0661_V3_LEFT, M0814_V3_LEFT, M0887_V0_LEFT,

M0904_V2_LEFT, M1246_V2_LEFT, M1362_V0_LEFT, M1947_V2_RIGHT, M1991_V1_RIGHT, M2181_V0_LEFT, M2307_V1_LEFT,

M2384_V0_RIGHT, M2800_V2_RIGHT, M2872_V2_RIGHT, M0187_V3_LEFT, M0193_V0_RIGHT, M0646_V0_LEFT, M1036_V0_RIGHT,

M1045_V2_RIGHT, M1186_V3_LEFT, M1320_V0_RIGHT, M1393_V0_LEFT, M1411_V2_LEFT, M1447_V0_LEFT, M1476_V0_LEFT,

M1487_V0_LEFT, M1665_V0_LEFT, M1797_V0_LEFT, M1849_V2_RIGHT, M1926_V0_RIGHT, M0371_V3_RIGHT, M0399_V3_LEFT,

M0720_V2_LEFT, M0857_V0_RIGHT, M2243_V3_LEFT, M2308_V0_LEFT, M2894_V0_RIGHT, M2920_V3_LEFT, M2964_V1_RIGHT,

M0413_V2_LEFT, M0766_V0_LEFT, M1177_V0_LEFT, M1268_V0_LEFT, M1394_V0_LEFT, M1458_V0_LEFT, M1585_V0_RIGHT,

M1684_V0_RIGHT, M1913_V3_LEFT, M2029_V0_RIGHT, M2050_V0_LEFT, M2067_V2_LEFT, M2430_V2_RIGHT, M2542_V0_LEFT,

M2788_V3_LEFT, M3006_V0_LEFT, M0241_V2_RIGHT, M0452_V0_LEFT, M0808_V1_LEFT, M0924_V0_LEFT, M0936_V2_LEFT,

M1476_V2_RIGHT, M1894_V3_RIGHT, M2361_V3_LEFT, M2569_V0_RIGHT, M2689_V1_LEFT, M2741_V2_RIGHT, M0303_V2_RIGHT,

M0453_V0_RIGHT, M0934_V0_RIGHT, M1375_V3_RIGHT, M1837_V2_LEFT, M1995_V3_RIGHT, M2373_V0_RIGHT, M2448_V0_RIGHT,

M2470_V0_LEFT, M2965_V0_RIGHT, M2995_V3_RIGHT, M0066_V3_LEFT, M0123_V3_LEFT, M0203_V1_RIGHT, M0540_V0_RIGHT, M0693_V2_RIGHT, M0707_V3_LEFT, M0861_V2_LEFT, M0954_V0_RIGHT, M1793_V0_RIGHT, M2014_V2_LEFT, M2281_V2_LEFT, M2353_V0_RIGHT, M2418_V0_LEFT, M0201_V2_RIGHT, M0324_V2_LEFT, M0504_V3_LEFT, M0541_V3_RIGHT, M0667_V1_LEFT, M1021_V2_RIGHT, M1291_V0_RIGHT, M1294_V1_LEFT, M1600_V0_LEFT, M1625_V1_LEFT, M1778_V2_RIGHT, M2510_V0_RIGHT, M2715_V2_RIGHT, M2775_V3_LEFT, M0090_V3_RIGHT, M0136_V3_LEFT, M0175_V2_RIGHT, M1674_V2_RIGHT, M1869_V2_LEFT, M2266_V2_RIGHT, M2356_V3_RIGHT, M0601_V3_RIGHT, M1448_V3_RIGHT, M1527_V2_LEFT, M1942_V3_RIGHT, M2031_V3_LEFT, M2150_V2_RIGHT, M2271_V3_LEFT, M2273_V2_LEFT, M2722_V2_RIGHT, M2732_V0_RIGHT, M2781_V2_RIGHT, M0767_V2_RIGHT, M0810_V2_RIGHT, M1243_V2_RIGHT, M1644_V0_LEFT, M1717_V2_RIGHT, M2568_V3_RIGHT, M2657_V0_RIGHT, M2848_V1_LEFT, M2900_V2_RIGHT, M2957_V2_RIGHT, M2998_V2_RIGHT, M0115_V0_RIGHT, M0453_V2_LEFT, M0578_V2_RIGHT, M0648_V3_LEFT, M1064_V2_LEFT, M1128_V2_LEFT, M1199_V0_LEFT, M1391_V2_RIGHT, M1851_V0_LEFT, M1926_V1_LEFT, M2080_V0_RIGHT, M2341_V2_LEFT, M2778_V1_LEFT, M0011_V2_RIGHT, M0071_V0_RIGHT, M0408_V3_LEFT, M0687_V0_RIGHT, M0714_V0_LEFT, M0951_V0_LEFT, M0994_V3_RIGHT, M1202_V3_RIGHT, M1516_V3_LEFT, M1617_V3_RIGHT, M1779_V0_RIGHT, M2490_V2_LEFT, M2715_V0_LEFT, M2855_V2_LEFT, M3021_V2_LEFT, M0008_V0_LEFT, M0224_V0_RIGHT, M0226_V0_LEFT, M0566_V2_RIGHT, M0696_V0_RIGHT, M0981_V0_LEFT, M1243_V1_LEFT, M1279_V0_RIGHT, M1339_V2_RIGHT, M1512_V2_LEFT, M1691_V3_RIGHT, M2192_V2_LEFT, M2754_V2_LEFT, M3018_V3_RIGHT, M0479_V3_LEFT, M0550_V2_LEFT, M0808_V3_RIGHT, M0961_V2_RIGHT, M1007_V0_LEFT, M1198_V2_LEFT, M1507_V0_LEFT, M1735_V3_RIGHT, M2464_V0_LEFT, M2556_V3_RIGHT, M2657_V2_LEFT, M0016_V2_LEFT, M0098_V2_RIGHT, M0124_V0_RIGHT, M0132_V0_LEFT, M0273_V2_LEFT, M1287_V0_LEFT, M1360_V2_LEFT, M1466_V0_RIGHT, M1714_V0_RIGHT, M1745_V2_LEFT, M1804_V2_RIGHT, M1991_V2_RIGHT, M2072_V2_RIGHT, M2178_V0_RIGHT, M2307_V2_LEFT, M2870_V2_LEFT, M0457_V2_RIGHT, M0571_V2_LEFT, M0646_V1_LEFT, M0691_V0_LEFT, M1184_V2_RIGHT, M1476_V1_LEFT, M1643_V2_LEFT, M1665_V1_LEFT, M2178_V2_LEFT, M2229_V2_RIGHT, M2650_V2_LEFT, M0167_V0_LEFT, M0193_V2_LEFT, M0205_V2_RIGHT, M0495_V0_RIGHT, M0617_V0_RIGHT, M0716_V2_RIGHT, M1475_V0_RIGHT, M1659_V2_LEFT, M1854_V2_RIGHT, M1942_V2_LEFT, M2678_V0_LEFT, M2750_V2_RIGHT, M2964_V2_RIGHT, M0448_V0_LEFT, M0877_V0_RIGHT, M1659_V0_RIGHT, M1707_V2_LEFT, M1812_V0_RIGHT, M2239_V0_LEFT, M2256_V2_LEFT, M2556_V0_LEFT, M2607_V2_RIGHT, M2716_V0_RIGHT, M2735_V2_LEFT, M2775_V0_RIGHT, M2879_V0_RIGHT, M2967_V0_RIGHT, M0566_V0_LEFT, M0808_V2_LEFT, M1390_V2_RIGHT, M1567_V2_RIGHT, M1733_V0_LEFT, M2159_V0_LEFT, M2689_V2_LEFT, M0032_V2_RIGHT, M0033_V2_LEFT, M0453_V1_RIGHT, M0999_V2_RIGHT, M1270_V0_RIGHT, M1681_V0_RIGHT, M3026_V2_RIGHT, M0203_V2_RIGHT, M0967_V2_LEFT, M0667_V2_LEFT, M0815_V2_RIGHT, M1233_V2_LEFT, M1294_V2_LEFT, M2768_V2_LEFT.

## MOST Database Radiographs (XRay) Data:

M0233_V0_RIGHT, M2183_V0_RIGHT, M0327_V0_LEFT, M2119_V0_LEFT, M2166_V0_LEFT, M0620_V0_LEFT, M1711_V0_LEFT, M1811_V0_LEFT, M0759_V0_LEFT, M2305_V0_RIGHT, M2259_V0_LEFT, M0638_V0_RIGHT, M2120_V0_RIGHT, M0734_V0_RIGHT, M1101_V0_RIGHT, M0372_V0_LEFT, M1005_V0_LEFT, M1980_V0_LEFT, M2334_V0_RIGHT, M0058_V0_LEFT, M0381_V0_RIGHT,

M1897_V0_LEFT, M2170_V0_RIGHT, M2286_V0_RIGHT, M0015_V0_LEFT, M0968_V0_LEFT, M1241_V0_LEFT, M1749_V0_RIGHT, M2423_V0_LEFT, M0053_V0_LEFT, M1238_V0_RIGHT, M2045_V0_LEFT, M0661_V0_LEFT, M1014_V0_RIGHT, M0187_V0_LEFT, M1186_V0_LEFT, M1402_V0_RIGHT, M1526_V0_LEFT, M1955_V0_LEFT, M0229_V0_RIGHT, M0371_V0_RIGHT, M0399_V0_LEFT, M1502_V0_RIGHT, M2183_V1_RIGHT, M2243_V0_LEFT, M2687_V0_LEFT, M2920_V0_LEFT, M0905_V0_RIGHT, M0938_V0_LEFT, M1299_V0_RIGHT, M1913_V0_LEFT, M2788_V0_LEFT, M0576_V0_LEFT, M1894_V0_RIGHT, M2225_V0_LEFT, M2361_V0_LEFT, M2446_V0_RIGHT, M2561_V0_RIGHT, M1104_V0_LEFT, M1375_V0_RIGHT, M1995_V0_RIGHT, M2995_V0_RIGHT, M0066_V0_LEFT, M0123_V0_LEFT, M1811_V1_LEFT, M1912_V0_RIGHT, M0504_V0_LEFT, M0541_V0_RIGHT, M2560_V0_LEFT, M0090_V0_LEFT, M0136_V0_LEFT, M0700_V0_RIGHT, M2356_V0_RIGHT, M0601_V0_RIGHT, M0638_V1_RIGHT, M1305_V0_RIGHT, M1448_V0_RIGHT, M2031_V0_LEFT, M2271_V0_LEFT, M2737_V0_RIGHT, M0100_V0_RIGHT, M0231_V0_RIGHT, M0723_V0_RIGHT, M1570_V0_RIGHT, M1656_V0_RIGHT, M2568_V0_RIGHT, M0044_V0_LEFT, M0648_V0_LEFT, M0907_V0_LEFT, M2323_V0_LEFT, M2849_V2_LEFT, M0058_V1_LEFT, M0408_V0_LEFT, M0707_V0_RIGHT, M0994_V0_RIGHT, M1202_V0_RIGHT, M1377_V0_LEFT, M1516_V0_LEFT, M1617_V0_RIGHT, M1897_V1_LEFT, M2264_V0_RIGHT, M2286_V1_RIGHT, M0968_V1_LEFT, M1691_V0_RIGHT, M2094_V0_RIGHT, M2415_V0_RIGHT, M3018_V0_RIGHT, M0479_V0_LEFT, M1735_V0_RIGHT, M2476_V0_RIGHT, M1045_V0_LEFT, M1151_V0_RIGHT, M2110_V0_RIGHT, M2731_V0_LEFT, M0331_V0_RIGHT, M0511_V0_LEFT, M1402_V1_RIGHT, M1836_V0_LEFT, M2339_V0_LEFT, M0233_V3_RIGHT, M0814_V0_RIGHT, M1631_V0_RIGHT, M1774_V0_LEFT, M1788_V0_RIGHT, M2183_V2_RIGHT, M0019_V0_RIGHT, M0327_V2_LEFT, M0799_V0_RIGHT, M0938_V1_LEFT, M1300_V0_RIGHT, M2124_V0_RIGHT, M1894_V1_RIGHT, M2119_V2_LEFT, M2166_V2_LEFT, M2606_V0_LEFT, M0333_V0_LEFT, M0658_V0_RIGHT, M1995_V1_RIGHT, M0123_V1_LEFT, M0620_V2_LEFT, M0964_V0_LEFT, M1423_V0_LEFT, M1711_V2_LEFT, M1811_V2_LEFT, M0759_V2_LEFT, M1648_V0_LEFT, M2305_V2_RIGHT, M2560_V1_LEFT, M2775_V1_LEFT, M0605_V0_LEFT, M1097_V0_LEFT, M1314_V0_RIGHT, M1457_V0_LEFT, M1507_V0_RIGHT, M2259_V2_LEFT, M2353_V2_LEFT, M2958_V0_LEFT, M2978_V0_RIGHT, M0134_V0_RIGHT, M0291_V0_RIGHT, M0638_V2_RIGHT, M1305_V1_RIGHT, M1467_V0_LEFT, M2081_V0_LEFT, M2120_V2_RIGHT, M0247_V0_RIGHT, M0723_V1_RIGHT, M0734_V2_RIGHT, M0958_V0_RIGHT, M1101_V2_RIGHT, M1549_V0_LEFT, M1979_V0_RIGHT, M2103_V0_LEFT, M2357_V0_RIGHT, M2568_V1_RIGHT, M2718_V0_RIGHT, M3001_V0_LEFT, M0372_V2_LEFT, M1005_V2_LEFT, M1980_V2_LEFT, M2334_V2_RIGHT, M2839_V0_LEFT, M0058_V2_LEFT, M0381_V2_RIGHT, M0774_V0_RIGHT, M1897_V2_LEFT, M2170_V2_RIGHT, M2817_V0_LEFT, M0015_V2_LEFT, M0101_V0_RIGHT, M0968_V2_LEFT, M1241_V2_LEFT, M1486_V0_LEFT, M1749_V2_RIGHT, M2423_V2_LEFT, M0053_V2_LEFT, M0434_V0_RIGHT, M1238_V2_RIGHT, M1641_V0_RIGHT, M2045_V2_LEFT, M0529_V0_LEFT, M0661_V2_LEFT, M0904_V0_LEFT, M1014_V2_RIGHT, M1246_V0_LEFT, M1947_V0_RIGHT, M2800_V0_RIGHT, M2872_V0_RIGHT, M0187_V2_LEFT, M0540_V2_LEFT, M1186_V2_LEFT, M1402_V2_RIGHT, M1411_V0_LEFT, M1526_V2_LEFT, M1548_V0_LEFT, M1849_V0_RIGHT, M1955_V2_LEFT, M2339_V1_LEFT, M0229_V2_RIGHT, M0371_V2_RIGHT, M0399_V2_LEFT, M0720_V0_LEFT, M1502_V2_RIGHT, M2243_V2_LEFT, M2687_V2_LEFT, M2920_V2_LEFT, M0413_V0_LEFT, M0713_V0_RIGHT, M0799_V1_RIGHT, M0938_V2_LEFT, M1299_V2_RIGHT, M1572_V0_RIGHT, M1913_V2_LEFT, M2067_V0_LEFT, M2430_V0_RIGHT, M0241_V0_RIGHT, M0576_V2_LEFT, M0936_V0_LEFT,

M1894_V2_RIGHT, M2361_V2_LEFT, M2446_V2_RIGHT, M2561_V2_RIGHT, M2741_V0_RIGHT, M0303_V0_RIGHT, M1104_V2_LEFT,

M1375_V2_RIGHT, M1837_V0_LEFT, M1995_V2_RIGHT, M2995_V2_RIGHT, M0066_V2_LEFT, M0123_V2_LEFT, M0693_V0_RIGHT,

M0861_V0_LEFT, M1912_V2_RIGHT, M2014_V0_LEFT, M2281_V0_LEFT, M0201_V0_RIGHT, M0324_V0_LEFT, M0504_V2_LEFT,

M0541_V2_RIGHT, M1021_V0_RIGHT, M1778_V0_RIGHT, M2541_V0_RIGHT, M2560_V2_LEFT, M2775_V2_LEFT, M0090_V2_LEFT,

M0136_V2_LEFT, M0175_V0_RIGHT, M0700_V2_RIGHT, M1674_V0_RIGHT, M1869_V0_LEFT, M2266_V0_RIGHT, M2356_V2_RIGHT,

M0291_V1_RIGHT, M0601_V2_RIGHT, M0903_V2_RIGHT, M1448_V2_RIGHT, M1527_V0_LEFT, M2031_V2_LEFT, M2150_V0_RIGHT,

M2271_V2_LEFT, M2273_V0_LEFT, M2569_V2_LEFT, M2737_V2_RIGHT, M2781_V0_RIGHT, M0100_V2_RIGHT, M0231_V2_RIGHT,

M0723_V2_RIGHT, M0767_V0_RIGHT, M0810_V0_RIGHT, M1570_V2_RIGHT, M1656_V2_RIGHT, M1717_V0_RIGHT, M2568_V2_RIGHT,

M2900_V0_RIGHT, M2957_V0_RIGHT, M2998_V0_RIGHT, M3001_V1_LEFT, M0044_V2_LEFT, M0578_V0_RIGHT, M0648_V2_LEFT,

M0907_V2_LEFT, M1064_V0_LEFT, M1128_V0_LEFT, M1391_V0_RIGHT, M2323_V2_LEFT, M2341_V0_LEFT, M0011_V0_RIGHT,

M0408_V2_LEFT, M0707_V2_RIGHT, M0994_V2_RIGHT, M1120_V0_LEFT, M1202_V2_RIGHT, M1377_V2_LEFT, M1516_V2_LEFT,

M1617_V2_RIGHT, M2264_V2_RIGHT, M2308_V2_RIGHT, M2490_V0_LEFT, M2728_V0_LEFT, M2855_V0_LEFT, M3021_V0_LEFT,

M1339_V0_RIGHT, M1512_V0_LEFT, M1691_V2_RIGHT, M2192_V0_LEFT, M2415_V2_RIGHT, M2754_V0_LEFT, M0479_V2_LEFT,

M0512_V0_LEFT, M0550_V0_LEFT, M0961_V0_RIGHT, M1198_V0_LEFT, M1735_V2_RIGHT, M2476_V2_RIGHT, M2556_V2_RIGHT,

M0016_V0_LEFT, M0098_V0_RIGHT, M0163_V0_RIGHT, M0264_V0_LEFT, M0273_V0_LEFT, M0904_V1_LEFT, M1045_V2_LEFT,

M1151_V2_RIGHT, M1360_V0_LEFT, M1362_V2_RIGHT, M1745_V0_LEFT, M1804_V0_RIGHT, M1991_V0_RIGHT, M2072_V0_RIGHT,

M2110_V2_RIGHT, M2307_V0_LEFT, M2731_V2_LEFT, M2870_V0_LEFT, M0319_V0_RIGHT, M0331_V2_RIGHT, M0457_V0_RIGHT,

M0511_V2_LEFT, M0571_V0_LEFT, M0899_V0_LEFT, M1184_V0_RIGHT, M1533_V0_RIGHT, M1836_V2_LEFT, M2229_V0_RIGHT,

M2339_V2_LEFT, M2650_V0_LEFT, M0205_V0_RIGHT, M0716_V0_RIGHT, M0814_V2_RIGHT, M1171_V0_LEFT, M1631_V2_RIGHT,

M1774_V2_LEFT, M1788_V2_RIGHT, M1942_V0_LEFT, M2183_V3_RIGHT, M2750_V0_RIGHT, M2964_V0_RIGHT, M0019_V2_RIGHT,

M0327_V3_LEFT, M0799_V2_RIGHT, M1237_V0_RIGHT, M1300_V2_RIGHT, M1393_V2_RIGHT, M1707_V0_LEFT, M2067_V1_LEFT,

M2124_V2_RIGHT, M2256_V0_LEFT, M2607_V0_RIGHT, M2735_V0_LEFT, M0808_V0_LEFT, M1567_V0_RIGHT, M2119_V3_LEFT,

M2689_V0_LEFT, M0032_V0_RIGHT, M0033_V0_LEFT, M0333_V2_LEFT, M0658_V2_RIGHT, M0999_V0_RIGHT, M1834_V0_LEFT,

M1948_V0_RIGHT, M0203_V0_RIGHT, M0964_V2_LEFT, M0967_V0_LEFT, M1423_V2_LEFT, M1711_V3_LEFT, M1811_V3_LEFT,

M2347_V0_RIGHT, M0667_V0_LEFT, M0716_V3_LEFT, M0815_V0_RIGHT, M1021_V1_RIGHT, M1233_V0_LEFT, M1294_V0_LEFT,

M1625_V0_LEFT, M1648_V2_LEFT, M2048_V0_LEFT, M2305_V3_RIGHT, M2384_V3_LEFT, M2442_V0_LEFT, M2552_V0_LEFT,

M2715_V1_RIGHT, M2744_V0_RIGHT, M2768_V0_LEFT, M0268_V0_LEFT, M0605_V2_LEFT, M0903_V0_LEFT, M1314_V2_RIGHT,

M1457_V2_LEFT, M1507_V2_RIGHT, M2259_V3_LEFT, M2266_V1_RIGHT, M2353_V3_LEFT, M2958_V2_LEFT, M2978_V2_RIGHT,

M3026_V0_LEFT, M0134_V2_RIGHT, M0176_V0_RIGHT, M0291_V2_RIGHT, M0638_V3_RIGHT, M1467_V2_LEFT, M1497_V0_RIGHT,

M2081_V2_LEFT, M2120_V3_RIGHT, M2270_V0_LEFT, M0247_V2_RIGHT, M0734_V3_RIGHT, M0844_V0_RIGHT, M0958_V2_RIGHT,

M1089_V0_LEFT, M1101_V3_RIGHT, M1549_V2_LEFT, M1979_V2_RIGHT, M2058_V0_RIGHT, M2103_V2_LEFT, M2357_V2_RIGHT,

M2718_V2_RIGHT, M2848_V0_LEFT, M2957_V1_RIGHT, M3001_V2_LEFT, M0578_V1_RIGHT, M1005_V3_LEFT, M1340_V0_RIGHT,

M1965_V0_RIGHT, M1980_V3_LEFT, M2022_V0_RIGHT, M2334_V3_RIGHT, M2778_V0_LEFT, M2839_V2_LEFT, M0058_V3_LEFT,

M0774_V2_RIGHT, M1747_V0_LEFT, M1897_V3_LEFT, M2170_V3_RIGHT, M2817_V2_LEFT, M0015_V3_LEFT, M0827_V0_RIGHT,

M0968_V3_LEFT, M1177_V2_RIGHT, M1243_V0_LEFT, M1486_V2_LEFT, M1643_V0_RIGHT, M1684_V2_LEFT, M1747_V2_RIGHT,

M1749_V3_RIGHT, M1835_V0_LEFT, M2423_V3_LEFT, M2849_V0_RIGHT, M0022_V0_RIGHT, M0053_V3_LEFT, M0434_V2_RIGHT,

M0470_V0_LEFT, M0550_V1_LEFT, M0588_V0_RIGHT, M1198_V1_LEFT, M1238_V3_RIGHT, M1604_V0_RIGHT, M1641_V2_RIGHT,

M0098_V1_RIGHT, M0264_V1_LEFT, M0529_V2_LEFT, M0661_V3_LEFT, M0887_V0_LEFT, M0904_V2_LEFT, M1014_V3_RIGHT,

M1246_V2_LEFT, M1362_V0_LEFT, M1947_V2_RIGHT, M2181_V0_LEFT, M2307_V1_LEFT, M2384_V0_RIGHT, M2800_V2_RIGHT,

M2872_V2_RIGHT, M0187_V3_LEFT, M0193_V0_RIGHT, M0646_V0_LEFT, M1036_V0_RIGHT, M1186_V3_LEFT, M1320_V0_RIGHT,

M1393_V0_LEFT, M1411_V2_LEFT, M1447_V0_LEFT, M1476_V0_LEFT, M1487_V0_LEFT, M1526_V3_LEFT, M1665_V0_LEFT,

M1797_V0_LEFT, M1849_V2_RIGHT, M1926_V0_RIGHT, M0229_V3_RIGHT, M0371_V3_RIGHT, M0399_V3_LEFT, M0720_V2_LEFT,

M0857_V0_RIGHT, M1502_V3_RIGHT, M2243_V3_LEFT, M2308_V0_LEFT, M2894_V0_RIGHT, M2920_V3_LEFT, M2964_V1_RIGHT,

M0413_V2_LEFT, M0713_V2_RIGHT, M0766_V0_LEFT, M1177_V0_LEFT, M1268_V0_LEFT, M1394_V0_LEFT, M1458_V0_LEFT,

M1572_V2_RIGHT, M1684_V0_RIGHT, M1913_V3_LEFT, M2029_V0_RIGHT, M2050_V0_LEFT, M2067_V2_LEFT, M2430_V2_RIGHT,

M2542_V0_LEFT, M2788_V3_LEFT, M3006_V0_LEFT, M0241_V2_RIGHT, M0452_V0_LEFT, M0576_V3_LEFT, M0924_V0_LEFT,

M0936_V2_LEFT, M1476_V2_RIGHT, M1894_V3_RIGHT, M2361_V3_LEFT, M2569_V0_RIGHT, M2689_V1_LEFT, M2741_V2_RIGHT,

M0303_V2_RIGHT, M0453_V0_RIGHT, M0934_V0_RIGHT, M1375_V3_RIGHT, M1834_V1_LEFT, M1837_V2_LEFT, M1995_V3_RIGHT,

M2373_V0_RIGHT, M2448_V0_RIGHT, M2470_V0_LEFT, M2965_V0_RIGHT, M2995_V3_RIGHT, M0066_V3_LEFT, M0123_V3_LEFT,

M0203_V1_RIGHT, M0540_V0_RIGHT, M0693_V2_RIGHT, M0954_V0_RIGHT, M1793_V0_RIGHT, M1912_V3_RIGHT, M2014_V2_LEFT,

M2281_V2_LEFT, M2353_V0_RIGHT, M2418_V0_LEFT, M0201_V2_RIGHT, M0324_V2_LEFT, M0504_V3_LEFT, M0541_V3_RIGHT,

M0667_V1_LEFT, M1021_V2_RIGHT, M1294_V1_LEFT, M1600_V0_LEFT, M1625_V1_LEFT, M1641_V3_LEFT, M1778_V2_RIGHT,

M2510_V0_RIGHT, M2560_V3_LEFT, M2715_V2_RIGHT, M2775_V3_LEFT, M0090_V3_LEFT, M0136_V3_LEFT, M0175_V2_RIGHT,

M1674_V2_RIGHT, M1869_V2_LEFT, M2266_V2_RIGHT, M2356_V3_RIGHT, M0601_V3_RIGHT, M1448_V3_RIGHT, M1527_V2_LEFT,

M1942_V3_RIGHT, M2031_V3_LEFT, M2150_V2_RIGHT, M2271_V3_LEFT, M2273_V2_LEFT, M2732_V0_RIGHT, M2781_V2_RIGHT,

M0100_V3_RIGHT, M0767_V2_RIGHT, M0810_V2_RIGHT, M1243_V2_RIGHT, M1644_V0_LEFT, M1717_V2_RIGHT, M2568_V3_RIGHT,

M2657_V0_RIGHT, M2900_V2_RIGHT, M2957_V2_RIGHT, M2998_V2_RIGHT, M0115_V0_RIGHT, M0453_V2_LEFT, M0578_V2_RIGHT,

M0648_V3_LEFT, M1064_V2_LEFT, M1128_V2_LEFT, M1199_V0_LEFT, M1391_V2_RIGHT, M1851_V0_LEFT, M2080_V0_RIGHT,

M2183_V5_RIGHT, M2341_V2_LEFT, M0011_V2_RIGHT, M0071_V0_RIGHT, M0408_V3_LEFT, M0687_V0_RIGHT, M0707_V3_RIGHT,

M0714_V0_LEFT, M0951_V0_LEFT, M0994_V3_RIGHT, M1120_V2_LEFT, M1202_V3_RIGHT, M1377_V3_LEFT, M1516_V3_LEFT,

M1617_V3_RIGHT, M1779_V0_RIGHT, M2490_V2_LEFT, M2715_V0_LEFT, M2728_V2_LEFT, M2855_V2_LEFT, M3021_V2_LEFT,

M0008_V0_LEFT, M0224_V0_RIGHT, M0226_V0_LEFT, M0566_V2_RIGHT, M0696_V0_RIGHT, M0981_V0_LEFT, M1243_V1_LEFT,

M1279_V0_RIGHT, M1339_V2_RIGHT, M1512_V2_LEFT, M1691_V3_RIGHT, M2119_V5_LEFT, M2192_V2_LEFT, M2415_V3_RIGHT, M2754_V2_LEFT, M3018_V3_RIGHT, M0479_V3_LEFT, M0512_V2_LEFT, M0550_V2_LEFT, M0808_V3_RIGHT, M0961_V2_RIGHT, M1007_V0_LEFT, M1198_V2_LEFT, M1735_V3_RIGHT, M2464_V0_LEFT, M2556_V3_RIGHT, M2657_V2_LEFT, M0016_V2_LEFT, M0098_V2_RIGHT, M0124_V0_RIGHT, M0132_V0_LEFT, M0163_V2_RIGHT, M0273_V2_LEFT, M1151_V3_RIGHT, M1287_V0_LEFT, M1360_V2_LEFT, M1466_V0_RIGHT, M1711_V5_LEFT, M1714_V0_RIGHT, M1745_V2_LEFT, M1804_V2_RIGHT, M1811_V5_LEFT, M1991_V2_RIGHT, M2072_V2_RIGHT, M2178_V0_RIGHT, M2307_V2_LEFT, M2870_V2_LEFT, M0331_V3_RIGHT, M0457_V2_RIGHT, M0571_V2_LEFT, M0646_V1_LEFT, M0691_V0_LEFT, M0899_V2_LEFT, M1184_V2_RIGHT, M1476_V1_LEFT, M1533_V2_RIGHT, M1643_V2_LEFT, M1665_V1_LEFT, M1836_V3_LEFT, M2178_V2_LEFT, M2229_V2_RIGHT, M2650_V2_LEFT, M0167_V0_LEFT, M0193_V2_LEFT, M0205_V2_RIGHT, M0495_V0_RIGHT, M0617_V0_RIGHT, M0716_V2_RIGHT, M0814_V3_RIGHT, M1171_V2_LEFT, M1475_V0_RIGHT, M1631_V3_RIGHT, M1659_V2_LEFT, M1774_V3_LEFT, M1942_V2_LEFT, M2678_V0_LEFT, M2750_V2_RIGHT, M2964_V2_RIGHT, M0448_V0_LEFT, M0799_V3_RIGHT, M0877_V0_RIGHT, M1659_V0_RIGHT, M1707_V2_LEFT, M1812_V0_RIGHT, M2256_V2_LEFT, M2556_V0_LEFT, M2607_V2_RIGHT, M2716_V0_RIGHT, M2735_V2_LEFT, M2775_V0_RIGHT, M2879_V0_RIGHT, M2967_V0_RIGHT, M0566_V0_LEFT, M0808_V2_LEFT, M1567_V2_RIGHT, M1733_V0_LEFT, M2159_V0_LEFT, M2689_V2_LEFT, M0032_V2_RIGHT, M0033_V2_LEFT, M0453_V1_RIGHT, M0658_V3_RIGHT, M0999_V2_RIGHT, M1005_V5_LEFT, M1270_V0_RIGHT, M1681_V0_RIGHT, M1834_V2_LEFT, M1980_V5_LEFT, M2334_V5_RIGHT, M3026_V2_RIGHT, M0058_V5_LEFT, M0203_V2_RIGHT, M0381_V5_RIGHT, M0967_V2_LEFT, M1423_V3_LEFT, M2170_V5_RIGHT, M0015_V5_LEFT, M0667_V2_LEFT, M0815_V2_RIGHT, M1233_V2_LEFT, M1294_V2_LEFT, M1749_V5_RIGHT, M2423_V5_LEFT, M2552_V2_LEFT, M2768_V2_LEFT.
